# Very early life microbiome and metabolome correlates with primary vaccination variability in children

**DOI:** 10.1128/msystems.00661-23

**Published:** 2023-08-23

**Authors:** Michael Shaffer, Katharine Best, Catherine Tang, Xue Liang, Steven Schulz, Eduardo Gonzalez, Cory H. White, Thomas P. Wyche, John Kang, Hendrik Wesseling, Begüm D. Topçuoğlu, Thomas Cairns, Theodore R. Sana, Robin M. Kaufhold, Julia M. Maritz, Christopher H. Woelk, Gokul Swaminathan, James E. Norton, Michael E. Pichichero

**Affiliations:** 1 Exploratory Science Center, Merck & Co., Inc., Cambridge, Massachusetts, USA; 2 Rochester General Hospital Research Institute, Center for Infectious Diseases and Immunology, Rochester, New York, USA; 3 Infectious Diseases and Vaccine Research, MRL, Merck & Co., Inc., West Point, Pennsylvania, USA; Institute for Systems Biology, Seattle, Washington, USA

**Keywords:** microbiome, metabolome, vaccine response, antibiotics, children

## Abstract

Despite multiple vaccine doses early in life, a substantial proportion of infants do not mount protective responses. In this study, we followed a cohort of children over the first 2 years of life, collecting microbiome and metabolome data longitudinally to investigate correlates of lower and higher responses to primary vaccinations. We found that the stool and nasopharyngeal microbiome developed with age, though demonstrated divergent timing and patterns in maturation. When measured at child age 2 months, evenness of genera in the stool microbiome correlated with lower vaccine responses, upregulated metabolome genes that encode for lipid A biosynthesis and oxidative phosphorylation correlated with higher vaccine responses, and abundance of phenylpyruvic acid in serum correlated with lower vaccine responses, measured 10 months later. Antibiotic exposure was associated with low vaccine response, and microbiome/metabolome features at child age 2 months, before childhood vaccinations commenced, correlated with variations in vaccine responses measured at child age 1 year. These results indicate that there may be potential to intervene before first childhood vaccinations to improve later protection.

**IMPORTANCE:**

We show that simultaneous study of stool and nasopharyngeal microbiome reveals divergent timing and patterns of maturation, suggesting that local mucosal factors may influence microbiome composition in the gut and respiratory system. Antibiotic exposure in early life as occurs commonly, may have an adverse effect on vaccine responsiveness. Abundance of gut and/or nasopharyngeal bacteria with the machinery to produce lipopolysaccharide—a toll-like receptor 4 agonist—may positively affect future vaccine protection, potentially by acting as a natural adjuvant. The increased levels of serum phenylpyruvic acid in infants with lower vaccine-induced antibody levels suggest an increased abundance of hydrogen peroxide, leading to more oxidative stress in low vaccine-responding infants.

## INTRODUCTION

Vaccinations have been responsible for a remarkable reduction in disease and death. However, vaccine efficacy varies between individuals, and children in particular are prone to vaccine hyporesponsiveness, often mounting sub-protective antibody levels after vaccination (1). Variations in normal human gut microbiome composition have been linked to reduced efficacy in immune system-altering therapies, including response to checkpoint inhibitors (2, 3) and response to vaccines (4, 5). Antibiotics cause rapid shifts in microbiome composition. In mouse and non-human primate studies, antibiotic usage is associated with poorer response to vaccination (6). In human studies, disruption of the microbiome with antibiotics has been seen to result in reduced vaccine response (7, 8).

In infants, the composition of the gut microbiome changes rapidly in early life (9 – 11). Prior to weaning, microbes that consume milk sugars, such as *Bifidobacteria* and *Lactobacillus*, are abundant (12). After the diet switches to solid food and milk consumption is diminished, an adult microbiome profile develops (9), in which *Bacteroides* is most prevalent in Western societies (10, 11). Nasopharyngeal microbiome composition also develops in early life with *Staphylococcus* as the major member and low variation in beta diversity (13). Later in child life, *Streptococcus* and *Moraxella* increase in abundance (14, 15) and variation in beta diversity is greater. Additionally, the composition of the nasopharyngeal microbiome has been associated with disease susceptibility, including acute otitis media positivity, which is associated with *Haemophilus* abundance (16).

The potential influence of the gut microbiome on response to early childhood vaccines has recently been reviewed (17). Several studies have identified bacterial species in cohorts from low- and middle-income countries, including those in the *Bifidobacterium* and *Bacteroides* genera that may confer a vaccine response advantage, while *Streptococcus* is disadvantageous (18, 19). However, these studies have been limited by the difficulty in tracking the development of the microbiome longitudinally in children. A more complete understanding of how the microbiome and related host processes in early life influence the development of a protective vaccine response could lead to early intervention or enhanced vaccine efficacy.

In this study, we tracked the development of the gut and nasopharyngeal microbiome in early childhood, collecting host “omic” modality data and participant metadata including antibiotic use. Early recruitment, within the first weeks of life, allowed for assessment of factors in very early life that may influence vaccine response such as antibiotic exposure at or near birth, and longitudinal sampling allowed for an in-depth analysis of the development of the microbiome with age. In particular, we found that abundance of microbial genes involved in the lipopolysaccharide production and oxidative phosphorylation at 2 months, but not later in life, was associated with vaccine response at 1 year. Similarly, the abundance of phenylpyruvic acid in sera at 2 months was associated with vaccine response at 1 year. These results indicate that there may be potential to intervene before the first childhood vaccinations to establish protection earlier in life.

## RESULTS

### Cohort description

**Fig 1:**
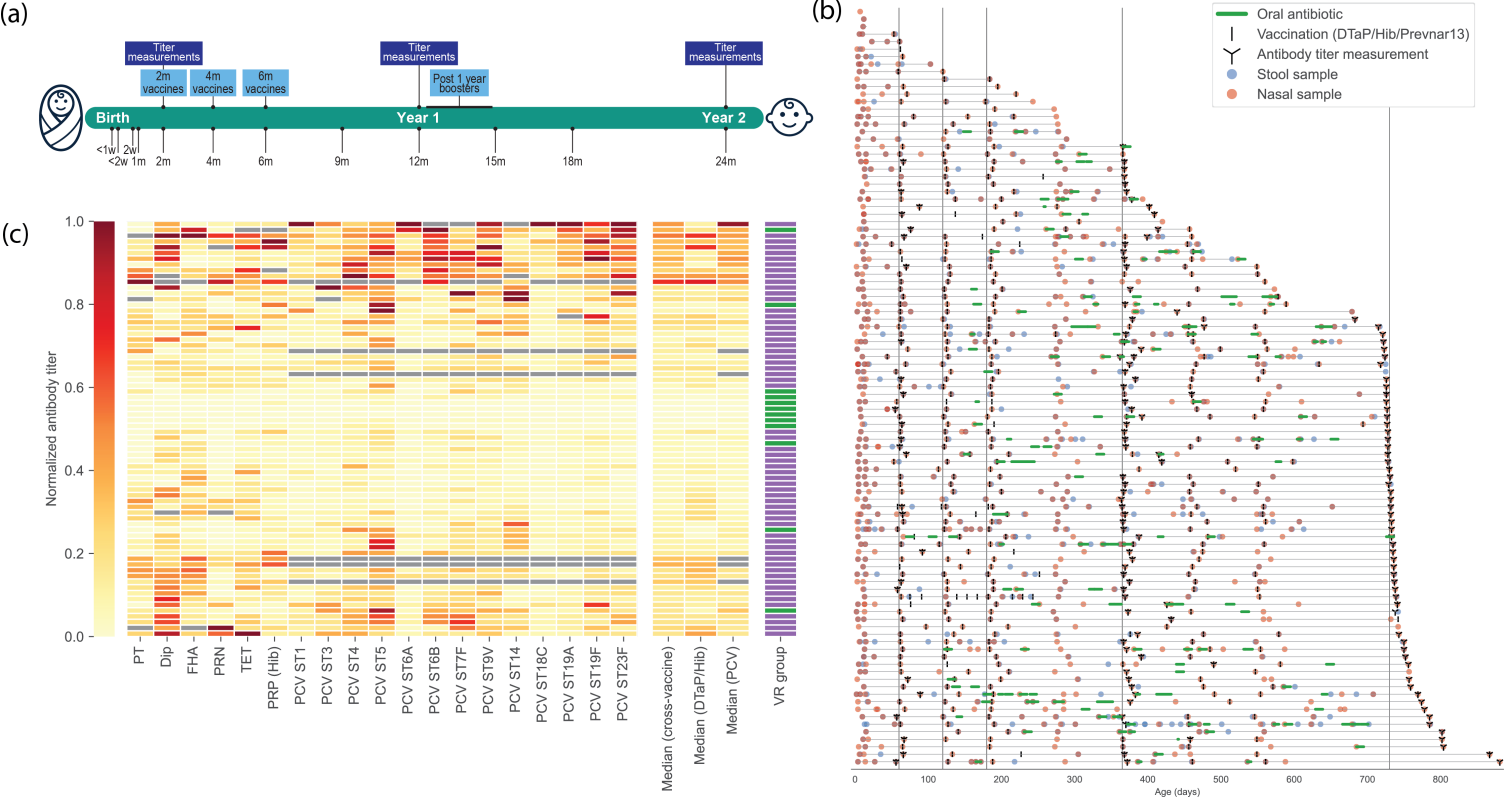
Longitudinal samples collected from children in the first 2 years of life to understand the relationships between antibiotic usage, microbiome, and vaccine response. (**a**) Study design. Subjects were recruited within 3 weeks of birth and followed through to 2 years. Blood samples were taken where possible at 2 months, 1 year, and 2 years to measure antibody response against vaccination antigens. Subjects were scheduled to attend well-check clinic visits regularly as shown at the bottom of the panel. Samples were collected as possible at these visits as well as any sick visits. (**b**) Longitudinal sampling from subjects. For each subject in the study, a timeline showing their time in the study by age in days (gray horizontal lines), stool and nasopharyngeal samples collected (red and blue circles), vaccinations received (black vertical lines), oral antibiotics taken (green horizontal bars), and blood samples taken to measure antibody responses (black antibody symbols). (**c**) Normalized antibody titers at 1 year. A normalized antibody titer was calculated for all 19 antigens measured in the study from 72 subjects at 1 year of age. Antigens measured were those included in the DTaP/Hib vaccine [diphtheria (DT), tetanus (TT), pertussis toxoid (PT), filamentous hemagglutinin (FHA), pertactin (PRN), and *Haemophilus influenzae* type b polysaccharide (PRP)] and 13 serotypes of *Streptococcus pneumoniae* included in the Prevnar 13 pneumococcal conjugate vaccine (PCV) (serotypes 1, 3, 4, 5, 6A, 6B, 7F, 9V, 14, 18C, 19A, 19F, and 23F). Median normalized titers were also calculated across vaccines, for the DTap/Hib vaccine only, and for the PCV only, for normal vaccine responders (NVR) indicted in purple, and low vaccine responders (LVR) indicated in green.

A total of 101 subjects were recruited into this study within the first 3 weeks of life. Follow-up was planned to age 2 years (Fig. 1a). At enrollment and later clinic visits, metadata were collected including sex, birth height and weight, race, gestation, delivery type, siblings, pets, breastfeeding/weaning status, daycare attendance, antibiotic usage, family medical history, and smoking status of parents (Table 1; Table S1). There were 83 subjects who had clinical visits past 1 year. Subjects visited the clinic a median of 10 times, with one subject having their samples collected 39 times (Fig. 1b). Where possible, stool and nasal samples were collected at clinic visits for microbiome profiling and plasma/serum was collected for metabolome and proteome profiling.

**TABLE 1:** Cohort descriptive statistics

		All participants	
		*n*	%
Vaccine response at 1 year	LVR	12	12%
	NVR	60	59%
	None recorded	29	29%
Sex	Male	55	54%
	Female	46	46%
Race	White/Caucasian	72	71%
	Black	9	9%
	Other	20	20%
Delivery type	Vaginal	82	81%
	C-section	19	19%
Smokers at home	No	78	77%
	Yes, outside	10	10%
	Yes	13	13%
Siblings	Yes	66	65%
	No	34	34%
	Not documented	1	1%
Pets at home	Yes	71	70%
	No	30	30%
Breastfed at enrollment	Yes, >50%	76	75%
	Yes, <50%	8	8%
	No	16	16%
	Not documented	1	1%
Breastfed at 6 months	Yes, >50%	38	38%
	Yes, <50%	4	4%
	No	47	47%
	Not documented	12	12%
Daycare at 6 months	Yes	20	20%
	No	69	68%
	Not documented	12	12%
Family history of ear infections	Yes	55	54%
	No	46	46%
Family history of other infections	Yes	20	20%
	No	81	80%
Family history of atopy	Yes	49	49%
	No	52	51%
		**Median**	**Inter-quartile Range (IQR)**
Age at enrollment	(days)	6	4–11
Height at birth (*n* = 87 documented)	(cm)	50.8	49.5–52.7
Weight at birth (*n* = 96 documented)	(kg)	3.5	3.1–3.9
Gestation (*n* = 100 documented)	(weeks)	39.6	39–40.3

A primary outcome of this study was vaccine response at 1 or 2 years of life. Previous literature defined normal vaccine responders (NVR) and low vaccine responders (LVR) based on the established or presumed protective thresholds against each of the six antigens included in the DTaP/Hib vaccine (20). Of the 101 subjects, we were able to assess the vaccination status of 72 subjects. Using the previous criteria, we found that 12 of the 72 participants (16.7%) were LVR at year 1 (Fig. 1c), in line with previously published results, and 2 of 56 participants (3.5%) were LVR at year 2, in line with expectations that achievement of protective antibody levels improved after post-1-year booster doses.

### Stool and nasal microbiome develop with age

To estimate the effects of the microbiome on vaccine response, the gut and nasopharynx microbiome were both characterized. Nasopharynx and stool samples were collected where possible at nine set time points throughout the first year of life, as well as three additional time points before 2 years of age. Additionally, when a subject visited the clinic for an illness, samples were collected as appropriate. Overall, 709 stool samples were collected. A median of seven stool samples was collected per subject, with one subject contributing 29 stool samples and five subjects without any associated stool samples. From the nasopharynx, 1,008 samples were collected with a median of 10 samples per subject and a maximum of 18 samples from a single subject. All subjects provided at least one nasopharynx sample.

**Fig 2:**
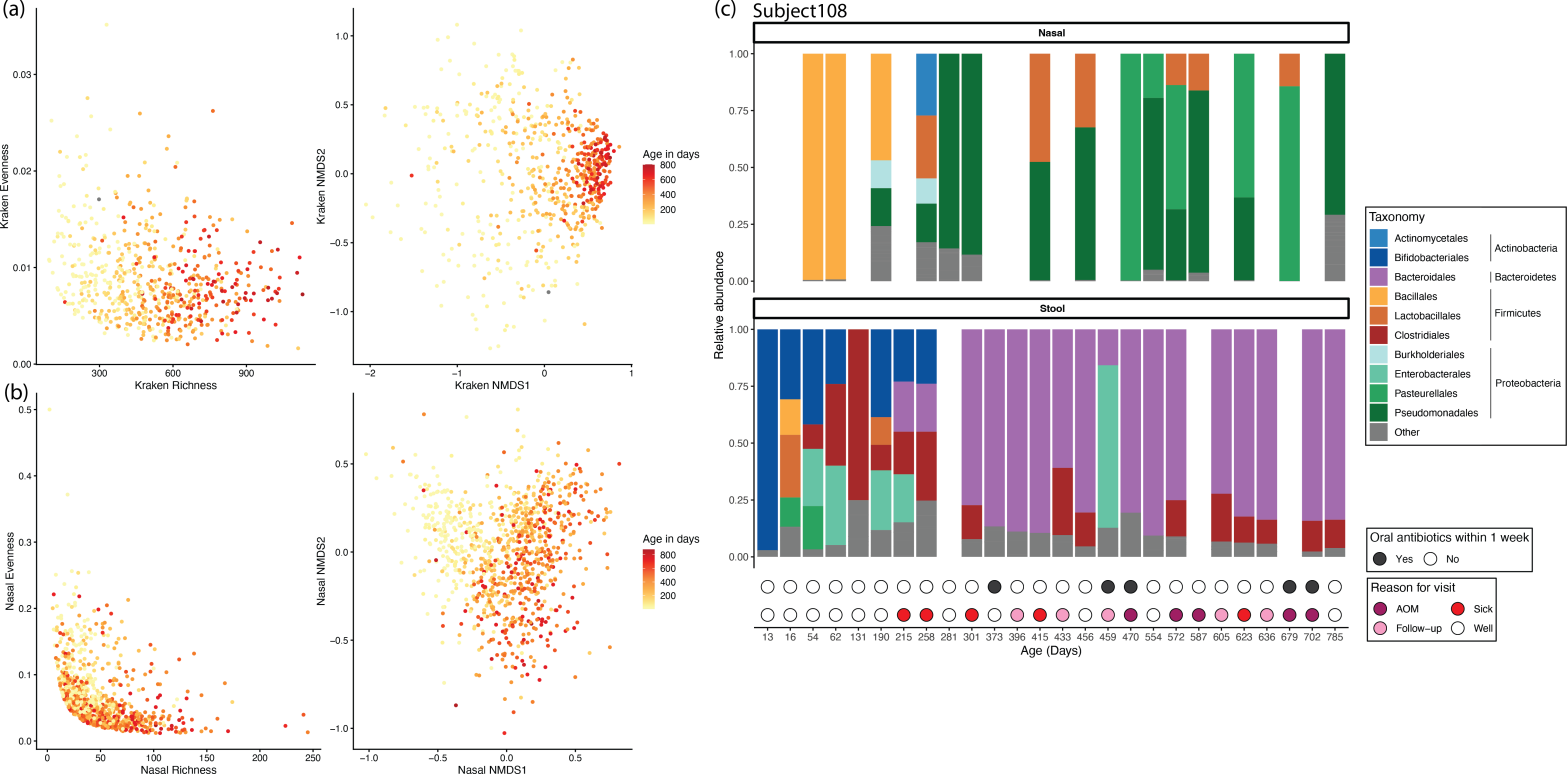
Longitudinal sampling allows for investigation of microbiome development with age. (**a**) Stool microbiome development. The Kraken genus alpha (left) and beta (right) diversity colored by the age of the subject when the sample was collected. (**b**) Nasal microbiome development. The nasal operational taxonomic unit (OTU) alpha (left) and beta (right) diversity colored by the age of the subject when the sample was collected. (**c**) Dense sampling from a study subject. Taxa bar plots of nasal (relative OTU abundance grouped by taxonomic order, top) and stool (relative Kraken order abundance, bottom) microbiome samples from subject 108 by age in days. Circles at the bottom indicate whether oral antibiotics were taken within 7 days of the sample (top row, black) and the type of clinic visit (pink-red circles, bottom).

Stool and nasopharynx samples were used to track the development of the microbial communities over time. There was clear development of the stool microbiome over time as measured via untargeted metagenomic sequencing (Fig. 2a). Richness, as measured via the number of genuses or Kyoto Encyclopedia of Genes and Genomes (KEGG) Orthology groups (KOs), increased with time, while evenness decreased with time (Fig. 2a, left) and converged on a common profile after 8 months. Bray-Curtis distances in the non-metric multidimensional scaling (NMDS) space (Fig. 2a, right) showed that stool microbial communities were highly diverse at early time points and became more similar over time. After the switch to solid food, most samples after 8 months of life clustered together (data not shown). To understand the function profiles of these samples, reads were mapped to KEGG genes and assigned KO IDs (see Materials and Methods). Similar changes in alpha and beta diversity over time were seen, as in the taxonomy data, albeit not as strong of an effect (Fig. S1).

The nasopharynx microbiome was characterized using 16S rRNA amplicon sequencing from nasal swabs. As with the stool samples, NMDS1 was strongly associated with age (Fig. 2b). The nasopharynx community started strongly clustered early in life but developed into a more diverse community with time. By 6 months of life, the samples stopped increasing in NMDS1 and became less variable along NMDS2, suggesting convergence to a common profile. Interestingly, the stool and nasal microbiome showed divergent patterns over time. In stool, richness increased with age, while there was no age-dependent pattern in the nasopharynx. Additionally, in stool, variability between subjects decreased with age, while in the nasopharynx communities diverged.

### Densely sampled subject demonstrates generalizable developmental trajectory

One subject (#108) was in the study for 785 days and visited the clinic 23 times. The very dense sampling allows for a detailed analysis of the development of the gut and nasopharynx microbiome (Fig. 2c; Supplemental Text).

### Response to DTaP/Hib vaccines and PCV

We sought to expand the LVR/NVR categorical analyses to evaluate if there was any association with continuous antibody measurements. Titer values were min-max transformed, as described in the Materials and Methods (Fig. S3). In addition to responses against DTaP/Hib antigens, we also measured responses against 13 *Streptococcus pneumoniae* serotypes included in the Prevnar 13 pneumococcal conjugate vaccine (PCV, Fig. 1c). We saw that the PCV antigen antibody measurements are more highly correlated with each other across participants than the DTaP/Hib antigen antibody measurements (Fig. S3b and c). When we looked at the min-max normalized antibody measurements for each child for all antigens considered in this study, we observed less separation between NVR and LVR than when only DTaP/Hib antigens were considered (Fig. S3d). As such, we considered vaccine response as both a categorical (NVR vs LVR) and continuous variable (median min-max normalized antibody titer across DTaP/Hib, PCV, or all antigens) in this study (Fig. 1c).

We observed a significant correlation between age at time of vaccination and vaccine response at year 1 (Fig. S4), which appeared to be driven by children who had their 2nd-month, 4th-month, and 6th-month doses later than scheduled, potentially due to the coronavirus disease 2019 (COVID-19) pandemic. There was no significant correlation between age at titer measurements at 1 year and measured vaccine response at 1 year. Additionally, we observed that, in general, antibody responses were correlated between year 1 and year 2 (Fig. S5).

### Antibiotic exposure

Across all participants in this study, we recorded prescriptions for 235 courses of antibiotics among 68 subjects (Table S2). Of these prescriptions, 173 (74%) were for oral antibiotics, intramuscular (IM) [3%], or intravenous (IV) [3%], with other prescriptions for topical antibiotics (20%) (Table S2). Since oral antibiotics made up most prescriptions in this cohort, we sought to assess only the effect of oral antibiotic usage during the first year of life, as previously observed and described (8). Two antibiotic use variables survived the multiplicity correction at the type I error control level at 0.1, following a double false discovery rate (FDR) procedure (21). First, among 72 children with available data at the time of birth, we found a negative association between antibiotic use at birth and vaccine response [prevalence of antibiotic use was higher in LVR (25%, 3 of 12) than in NVR (3.3%, 2 of 60) (Table S3), (pre-adjusted *P*-value = 0.03 and double FDR-adjusted *P*-value = 0.06)]. Second, the cumulative days of oral antibiotic use at 1 year was highly significantly associated with vaccine response (pre-adjusted *P*-value = 7.6 × 10^−8^ and double FDR-adjusted *P*-value = 9.1 × 10^−7^). Because there was a large percentage of babies who did not receive any oral antibiotics during the first year of life (47%), cumulative oral antibiotic use followed a zero inflated Poisson distribution. To better interpret the statistical significance from the omnibus likelihood ratio test, we visualized the difference of the oral antibiotic cumulative use between the LVR and NVR groups (Fig. S7). While the zero component (% of children not exposed to oral antibiotics at all) of the cumulative use at 1 year was not different between the two response groups (47% for NVR, and 50% for LVR), the non-zero count component (days of antibiotic exposure only among those infants who were exposed to oral antibiotics) was significantly higher (*P*-value = 9.1 × 10^−7^) in the LVR group (median non-zero antibiotic cumulative use = 28 days versus 10 days in LVR and NVR groups, respectively).

### Signals at 2 months, but not later vaccination time points, correlated with vaccine response at 1 year

With the hypothesis that microbiome state at time of vaccination impacts the ability of the immune system to respond appropriately, we tested for associations between diversity level measures of the stool and nasopharynx microbial communities and vaccine response. Alpha diversity was measured as richness, evenness, and Shannon diversity. After multiple test correction, the evenness of genera in the stool microbiome at 2 months was negatively correlated with DTaP/Hib vaccine response at child age 1 year (adjusted *P*-value = 0.04, Fig. S6). This correlation was not significant at other vaccination time points or at the time of vaccination. No other diversity measures from nasal operational taxonomic units (OTUs), stool KOs, or stool Kraken genera were significant. Additionally, no diversity measures were significantly associated with categorical vaccine response. At later vaccination time points (4 months and 6 months) as well as at time of outcome measurement (1 year), no diversity metrics were associated with any measure of vaccine response at 1 year. Similarly, no diversity metrics at any of these time points were associated with any measure of vaccine response at 2 years.

We next tested whether individual components of the microbiome at 2 months, 4 months, and 6 months of age were associated with vaccine response. None of the OTUs from the nasopharynx microbiome or taxa from the stool microbiome at any vaccination time points were correlated with any vaccine outcome variable at 1 or 2 years after multiple test correction.

**Fig 3:**
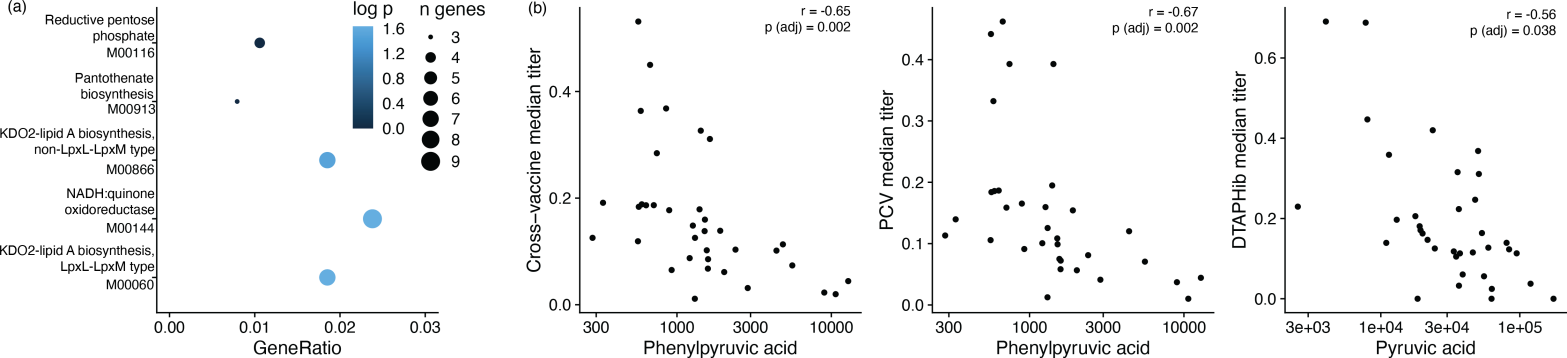
Early predictors of vaccine response identified in the 2-month microbiome and metabolome. (**a**) Enrichment analysis showing KO modules at 2 months associated with cross titer vaccine response at 1 year. KO module enrichment analysis was done to find modules significantly enriched (adjusted *P*-value <0.05) with KOs nominally significantly correlated with cross-vaccine titer (*P* < 0.05). Top hits are shown with x-axis showing the ratio of genes in the enriched module to the total number of genes in the analysis. (**b**) Correlations between metabolite abundances at 2 months and vaccine response at 1 year.

In the microbiome gene function (KEGG Orthology, KO) data, no KO abundances at vaccination time points were significantly associated with any vaccine response outcome metric at 1 year after multiple testing correction. However, at 2 months, many KOs had significant uncorrected *P*-values against vaccine response at 1 year (DTaP/Hib titer: 585, PCV titer: 262, cross-vaccine titer: 378, categorical response status: 32). These genes were used for an enrichment analysis with KEGG Modules. We found that three modules were enriched with gene functions positively correlated with cross-vaccine titer and two were enriched with gene functions correlated with median PCV titer (Fig. 3a). No modules were enriched with gene functions correlated with DTaP/Hib titer or with categorical response status. The modules associated with PCV were also positively associated with overall median titer (M00060 and M00866). Both are variants of the KD02-lipid A biosynthesis Raetz pathway and have seven genes with significant nominal *P*-values positively correlated with PCV and cross-vaccine titer (Fig. S8a). The additional module positively correlated with cross-vaccine titer (but not with PCV or DTaP/Hib titers separately) was an oxidoreductase (M00144, Fig. S8b). It should be noted that metagenomic sequencing was shallow (<1 gigabases) per sample after rarefying. It is possible that shallow sequencing may affect recall of individual genes and metabolic pathways.

The finding that features in the microbiome data at the time of first vaccination was associated with vaccine response led us to ask the same question of the metabolomics and proteomics data. For metabolomics, 111 compounds were detected in plasma or sera at 2 months (*n* = 36), 1 year (*n* = 52), 2 years (*n* = 44), and various other time points (*n* = 9). The abundance of phenylpyruvic acid in serum at 2 months was significantly negatively correlated with cross-vaccine and PCV titers, while pyruvic acid abundance in serum at 2 months was negatively correlated with DTaP/Hib titer (Fig. 3b). No significant findings were observed in the proteomics data at 2 months of age. We observed confounding across samples by age, and collection method (Fig. S10), and as such, these data were only able to be considered cross-sectionally rather than longitudinally.

Features from the microbiome and the metabolome at the time of earliest vaccination (2 months) were significantly correlated with continuous metrics of vaccine response at child age 1 year. Neither these features, nor any other features, at later vaccination time points (4 months and 6 months) were associated with vaccine response. Additionally, no features were seen to be associated with vaccination response at child age 2 years.

## DISCUSSION

Our cohort of children was enrolled within weeks of birth and followed over the first 2 years of life to explore the impact of the gut and nasopharyngeal microbiome as well as the metabolome and proteome on vaccination response. The stool and nasal microbiome data contribute to the body of work describing the development of the microbiome in early childhood.

The antibody response data collected build on previous findings regarding relationships between antibody titers against different vaccine antigens and allow us to consider the impact of timing of vaccine dose. In a previous study, children were categorized into clinically relevant low or normal vaccine responder groups based on protective thresholds against just six DTaP/Hib antigens (20). This performed equally well as using a larger panel of 13 antigens from five vaccines, suggesting broad correlation in response across vaccines. We considered 19 antibody titers against DTaP/Hib and PCV antigens and found that, in agreement with previous work, PCV titers were higher in children defined as NVR by DTaP/Hib responses than in children defined as LVR. Correlations were very strong within PCV antigens and weaker between PCV and DTaP/Hib antigens. While the LVR and NVR categories are clinically relevant, we found that continuous antibody titer provided more power to identify biological mechanism.

Exposure to antibiotics during the first days of life, as occurred in a small subset of infants in the study cohort (Table S3), may have had a particularly strong effect on vaccine responses because this is a highly sensitive period for nascent immune system development (22). Parenteral antibiotic use during early infancy is followed by incomplete recovery of the gut microbiota (23). Primary responses to antigens that have not been previously seen by the immune system may be more dependent on adjuvant signals from the microbiota. In the absence of pre-existing immunity, antibiotic-driven microbiota dysbiosis could lead to a significant impairment in the primary antibody response to vaccinations. In a study of 560 children (342 with and 218 without antibiotic prescriptions), vaccine-induced antibody levels following primary immunization to multiple vaccine antigens were lower in children given antibiotics (8). Here, we found that cumulative antibiotic exposures in the first year of life were associated with poor vaccine response measured at child age 1 year. It is possible that antibiotic use, via the effect on the microbiome, altered vaccine response. However, we were only able to determine an associative relationship between vaccine response and oral antibiotic usage. It is also possible that cumulative antibiotic usage could be a proxy for a subject with an immune system that is not able to respond well to vaccines or infectious organisms.

By using median antibody titer against antigens from each vaccine and across vaccines, we observed that higher abundance of genes which encode for lipid A biosynthesis in the gut at 2 months is associated with higher vaccine response at 1 year. Lipid A is the immunogenic subunit of lipopolysaccharide (LPS) [24], and previous research has shown how LPS exposure modulates immune responses in a number of settings (25 – 27). Additionally, monophosphoryl lipid A, a derivative of lipid A, is used as an adjuvant in Shingrix and other vaccines (28). Lipid A may modulate immune responses by interacting with antigen presenting cells via Toll-like receptor 4 (TLR4), instigating downstream immunomodulatory cytokine signaling pathways. Our data suggest that higher abundance of bacteria with the machinery to produce LPS could affect future vaccine protection potentially by acting as a natural adjuvant.

We additionally observed that the abundance of the module for the NADH:quinone oxidoreductase complex was associated with vaccine response at 1 year. All nominally significant genes from this module were positively correlated with vaccine response. Higher levels of oxidative phosphorylation could indicate a more oxidative environment in the gut which could suggest higher levels of inflammation (29). This difference in abundance may signal conditions which favor facultative anaerobes such as pathogenic proteobacteria. However, we were not able to find evidence that links these bacteria with stronger vaccine response.

In this study, increased levels of serum phenylpyruvic acid at 2 months were associated with lower cross-vaccine and PCV titers. Phenylpyruvic acid, ammonia, and hydrogen peroxide, a reactive oxygen species that causes oxidative stress, are byproducts of phenylalanine metabolism, which is catalyzed by the interleukin 4 (IL-4)-induced gene 1 (IL4I1) enzyme. Hydrogen peroxide leads to oxidative stress (30), which causes damage to mitochondria, a critical component of immune system regulation (31). The increased abundance of serum phenylpyruvic acid in infants with lower vaccine titers suggests an increased abundance of hydrogen peroxide, leading to more oxidative stress in these infants. IL4I1 (32), a secreted L-phenylalanine oxidase, has previously been shown to play a role in B and T cell adaptive immune responses, activate the aryl hydrocarbon receptor, be increased in cancer patients, and be involved in resistance to immune checkpoint inhibitor therapy in cancer patients (33). Consequently, IL4I1 is considered an immune checkpoint target, and based on our study as well as the role of IL4I1 in immune response in cancer, we hypothesize that IL4I1 and the hydrogen peroxide byproduct also play a role in vaccine response in infants. Phenylpyruvic acid and NADH:quinone oxidoreductase—though not in the same metabolic pathway—are both involved with oxidative stress, providing metabolite and gene-level support of this hypothesis (34 – 36).

It is notable that the key features associated with vaccine response at 1 year uncovered in this work were observed before the first vaccine dose. Additionally, the KO modules that were found to be enriched with nominally significant genes at 2 months were not enriched at 4 months or 6 months, even before multiple test correction. These results suggest that microbiome influences on later vaccine-induced protection occur very early in life. Additionally, this work has raised questions around the timing of protection in early childhood. We found features in very early life associated with vaccine response at 1 year, 6 months after the end of the primary course of vaccination doses, but not with vaccine response at 2 years, after booster doses. We therefore hypothesize that interventions in early life could enhance the timing of protection from childhood disease, allowing protective antibody levels to be established earlier.

There are inherent unavoidable limitations in this study due to the nature of the population. There was both an expected high level of subjects dropping out of the study and irregular timings across the cohort, which limited longitudinal analyses and complicated cross-sectional analyses. Limited blood volumes restricted possible analyses, and the appropriate method of blood collection is dependent on age. As a result, longitudinal analyses of data from blood were not pursued. Similarly, plasma was collected from some infants while serum was collected from others. Additionally, the processing of 16S rRNA sequencing data (nasopharynx microbiome) was conducted using methods that may overestimate diversity. Independent comparisons operating with mock community data have shown that clustering-based approaches may be prone to false positive OTUs (37).

This study set out to identify factors in the microbiome, metabolome, and proteome in early infancy associated with vaccine response at 1 or 2 years of age. In doing so, a rich longitudinal data set has been generated and is being made available to the research community for further analysis. We identified features from the microbiome and metabolome before the time of first vaccination that correlated with 1-year vaccine response. The timing of the observed important signals in our data suggests that up to 2 months may be a critical window of opportunity to intervene to have meaningful impact on the timing of vaccine-induced protection from childhood disease.

## MATERIALS AND METHODS

### Informed consent

Parents of all subjects enrolled in this study provided written oral consent for their participation herein. Study designs and endpoints were assessed and approved by an ethics committee and internal review board (IRB). This study has the IRB Protocol Number 1798.

### Subject recruitment, clinic visits, and sample collection

This was a prospective cohort study with enrollment within the first 3 weeks of life and follow-up to 2 years of age. The study population was derived from two primary care pediatric clinic practices in the Rochester Regional Health System (Bay Creek Pediatrics and Finger Lake Medical Associates-Pediatrics). Comprehensive demographic data and metadata were collected throughout the study. Enrollment of all children was completed in December 2018. Follow-up for 17 children aged 15–24 months was completed in March to December 2020, during the severe acute respiratory syndrome coronavirus 2 (COVID-19) pandemic. In a concurrently enrolled, separate cohort from the same study sites, the pandemic reduced the frequency of bacterial nasopharyngeal colonization and antibiotic use in 6- to 36-month-old subjects (38). The pandemic likely impacted occurrence of illnesses and antibiotic use among the 17 children, aged 15–24 months, who were still in follow-up during the pandemic.

All participants were due to receive regularly scheduled childhood vaccinations according to the US Centers for Disease Control schedule. Of relevance to this study, this included DTaP vaccine, Hib vaccine, and PCV at 2, 4, and 6 months of age and booster vaccines after 1 year of age. Dates of vaccinations were recorded for all children in the study.

The study design called for sample collection at child well-visits at ages 1, 2, and 3 weeks and then at 1, 2, 4, 6, 9, 12, 15, 18, and 24 months of age. When possible, sample collection included nasopharynx swabs, blood (for plasma or sera) via age-appropriate methods, and stool (collected either in-clinic or via at-home collection kits). Additionally, where children visited the clinic for an illness or a follow-up visit, the same samples were collected where feasible and appropriate.

Stool samples were collected either in-clinic or at home using DNA Genotek kits (DNA Genotek, Inc.), as per manufacturer instructions. If collected at home, samples are stored at −20°C (freezer), 4°C (fridge), or at room temperature for less than 2 weeks before storage and handling at −20°C. Nasopharynx swabs were collected in-clinic using either the ESwab Liquid Amies Collection and Transport System (Copan Diagnostics, Inc.) or Calgiswab Calcium Alginate Nasopharyngeal Swabs (Puritan Medical Products Co., LLC), as per manufacturer instructions. Plasma or sera samples were collected via heelstick, fingerstick, or venipuncture, depending on child age, parental agreement, and procedure success.

### Vaccine titer measurement, normalization, and vaccine response outcome variables

Antibody titers against six antigens included in the DTaP/Hib vaccine [diphtheria (DT), tetanus (TT), pertussis toxoid (PT), filamentous hemagglutinin (FHA), pertactin (PRN), and *Haemophilus influenzae* type b polysaccharide (PRP)] and 13 serotypes of *Streptococcus pneumoniae* included in the PCV (Pfizer, Inc.) (serotypes 1, 3, 4, 5, 6A, 6B, 7F, 9V, 14, 18C, 19A, 19F, and 23F) were measured at or around 1 y of age, and analysis in this study was focused on these responses. Additionally, for some participants, some antibody titers were measured at 2 months and 2 years of age. A total of 72 subjects had antibody measurement data at 1 year of age. For five of these participants, only the DTaP/Hib measurements, but not the PCV measurements, were accomplished. A total of 56 subjects had antibody measurement data at 2 years of age, all of whom had both DTaP/Hib and PCV antigen measurements.

Response to DTaP and Hib vaccination was assessed by measurement of total immunoglobulin G (IgG) antibody levels to DT, TT, PT, FHA, PRN and PRP antigens contained in these vaccines. The antibody levels were measured by enzyme-linked immunosorbent assays (ELISA), as previously described (39, 40). To assess response to PCV, serotype-specific pneumococcal capsular polysaccharide IgG antibodies were evaluated using a multiplexed electrochemiluminescence assay (41). IgG concentration was interpolated from the standard reference serum 007sp (42).

Outliers, defined as being more than three standard deviations away from the mean measurement for that antigen at the time point (1 year or 2 years of age), were removed. In total, this removed 30 of 1,303 antibody measurements at 1 year of age and 26 of 1,064 antibody measurements at 2 years of age.

As in previous studies (20), participants were classified as NVR or LVR based on their measurements compared to the established or presumed threshold of protection. Specifically, LVR is defined as having four or more titer values below protective thresholds (DT, TT, PRP, PT, PRN, and FHA below 0.1 IU/mL, 0.1 IU/mL, 0.15 µg/mL, 8 EU/mL, 8 IU/mL, and 8 IU/mL, respectively). Additionally, to take advantage of the PCV response data collected, we used continuous metrics of vaccine response. The measured antibody titers were min-max transformed within each antigen and time point, to ensure the same scale while maintaining the distributions of the data. For analysis, three continuous outcomes were defined: (i) median across all antigens (cross-vaccine titer), (ii) median across DTaP/Hib antigens, and (iii) median across PCV antigens.

### Nasal 16S rRNA sequencing

DNA was extracted from nasopharynx swabs using the QIAamp DNA Stool Mini Kit (Qiagen, Hilden, Germany), as per manufacturer instructions, from 1,008 samples. As controls, adult stool samples were processed in the same way. Mock communities (ATCC MSA-2002) were also included on all sequencing runs as controls. The V4 region of the 16S rRNA gene was amplified using the 515F and 806R 16S rRNA gene sequence primers (43). Amplified DNA was sequenced using the Illumina MiSeq platform with 2 × 250 bp paired-end reads, and resulting DNA sequences were processed as previously described (44). We used Mothur (v1.39.5) to merge paired-end reads (requiring complete overlap) and quality filter the sequences. Sequences were aligned to the SILVA database (v132), and chimeras as well as lineages of archaea, chloroplasts, and mitochondria were removed (45). OTUs were clustered at 97% similarity using OptiClust, and reads were aligned to OTUs to measure abundance. Before analysis, samples were rarefied to 10,000 reads per sample (944 samples passing threshold). To check for clustering compared to controls as well as by swab type and plate, we used NMDS with Bray-Curtis distance. None showed signs of batch effects (Fig. S9). Alpha diversity analyses were performed in python with diversity metrics calculated using scikit-bio (46). Beta diversity analyses were performed in *R* using vegan (47).

### Stool untargeted metagenomic sequencing

DNA was extracted from 709 stool samples using the Qiagen’s MagAttract PowerSoil DNA Kit (Qiagen, Hilden, Germany), as per manufacturer instructions. For untargeted metagenomic sequencing, genomic DNA was randomly amplified using the Illumina TruSeq library kit. As controls, adult stool samples, processed in the same way, as well as mock communities (ATCC MSA-2002) were included on all sequencing runs. Paired-end sequencing was done on the Illumina HiSeq using a universal primer in one direction and standard index primers in the reverse direction. Sequences were filtered for quality using bbduk for adapter removal and quality trimming (ktrim = 1, mink = 17, maq = 30, minlen = 75), and fastqc for general quality visualization (48). To remove reads from the PhiX and human genome, they were mapped with Bowtie2, and mapped reads were removed (49). Reads were then rarefied to 2.5 million reads per sample, and samples with less than 2.5 million reads were removed from the analysis (636 samples passing threshold). Metagenomic sequences were aligned to the KEGG database using DIAMOND (50, 51). KO counts were generated by compressing gene counts and equally splitting counts when genes were present in multiple KO groups, or vice versa. To determine the taxonomic content of the samples, KrakenUniq was used using default parameters (52).

For quality control (QC), we first checked for clustering compared to controls. Next, we checked for clustering within study samples by sequencing plate and collection location (at home vs in-clinic). No quality control issues were observed (Fig. S9). For functional and taxonomic data, alpha diversity analyses were performed in python with diversity metrics calculated using scikit-bio and beta diversity analyses were performed in *R* using vegan.

### Targeted metabolomics processing and data generation

Where sufficient blood sample volume was available, we generated metabolomics data as follows. An amount of 220 µL of acetonitrile was added to 55 µL plasma or sera in a microcentrifuge tube. The mixture was vortexed for 10 s and centrifuged for 10 min at 13,000 × *g* at 4°C. To remove residual larger proteins from the resuspended extract, 200 µL of the supernatant was transferred to a clean tube and dried by speedvac (Thermo Scientific Savant SPD2010). Each extract was resuspended in 100 µL water:methanol (90:10), transferred to a 0.2-µm Nanosep centrifugal filter (Pall #ODM02C35), and centrifuged for 10 min at 4°C at 13,000 × *g*. The flow-through was transferred to a 10-kDa Omega centrifugal filter (Pall #OD010C35) and centrifuged for 10 min at 4°C at 13,000 × *g*. The flow-through was transferred to a high-performance liquid chromatography (HPLC) vial and analyzed by targeted liquid chromatography-mass spectrometry (LC-MS/MS) analysis using three methods (Table S4 to S6) for the detection of central carbon metabolites and bile acids (Table S7). For each method, metabolites were detected using an Agilent 6470 Triple Quadrupole (QQQ) mass spectrometer coupled to an Agilent 1290 Infinity II HPLC. A QC sample, consisting of a pool of all samples, was injected multiple times during the sample acquisition sequence. Multiple reaction monitoring (MRM) transitions were determined from pure metabolite standards and the Agilent Metabolomics MRM Database and Method. All data were analyzed using Agilent Quantitative Analysis B.08 software.

Due to sample collection logistics, including age limitations on collection method, some samples used for metabolomics data generation were plasma and some were sera. Samples from the same time point were always the same sample type, e.g., serum at child age 2 months and plasma at child age 1 year. As such, analysis was done cross-sectionally.

### Proteomics processing and data generation

Where sufficient blood sample volume was available, we generated proteomics data as follows. Plasma and sera biofluid samples were processed using S-Trap sample processing. Therefore, 100 µg of sample was diluted in 5% SDS lysis buffer and reduced with 5 mM tris(2-carboxyethyl)phospine hydrochloride (TCEP) for 10 min at 37°C, alkylated with 100 mM iodoacetamide for 10 min at room temperature. Twelve percent phosphoric acid was added to 50 µL of SDS solubilized, reduced, and alkylated sample. A volume of 350 µL of S-Trap binding buffer (90% MeOH, 100 mM tetraethylammonium bromide [TEAB] final, pH 7.1) was added to the acidified lysate and the SDS lysate/S-Trap buffer mix was added to a 96-well spin column plate. Plates were spun in centrifuge for 30 s at 1,000 *g* or until all solution has passed through to bind proteins to the S-trap column and the flow-through removed.

Trapped proteins were washed by adding 400 µL S-Trap binding buffer for four times (350 µL for second and third wash, 175 µL for final wash). The flow-through was removed and collection plate changed for digestion. For digestion, Trypsin Gold (Promega Corporation) was added at 1:10–1:25 wt/wt in 125 µL of 50 mM TEAB, pH 8. The protease was briefly spun into the column; any solution that passed through to the top of the column was returned. It was ensured that there was no bubble atop the protein trap. The spin column was capped and incubated in a clean tube for ≥1 h at 47°C (for trypsin). Peptides were eluted with 80 µL each of 50 mM TEAB and 0.2% aqueous formic acid (FA) at 1,000 *g*. Hydrophobic peptides were eluted with 80 µL of 50% acetonitrile and 0.2% formic acid. Peptides were dried down and peptide levels were matched after digestion.

Dried down peptides were resuspended in 0.1% FA in H_2_O and loaded on Evotips according to the manufacturer’s protocol. LC-MS analysis was performed on an Evosep One liquid chromatography system interfaced with an Orbitrap QE-HFX (ThermoFisher Scientific) mass spectrometer. Peptides were separated by a linear 28 min gradient (Whisper 100, beta, 40 SPD) on a C18 analytical column (EV1112, 15 cm × 7.5 µm, ReproSil-Pur C18, 1.9 µm beads by Dr. Maisch) connected to a fused silica emitter (10 µm i.d., Evosep, EV-1111) and mounted on an EASY-Spray source (ThermoFisher Scientific). Mobile phases A and B were 0.1% FA in water and 0.1% FA in acetonitrile (ACN), respectively. A full mass spectrum with resolution of 60,000 [relative to a mass-to-charge (*m/z*) of 200] was acquired in a mass range of *m/z* 350–1,400 (automatic gain control [AGC] target 3 × 10^6^, maximum injection time 50 ms). The 30 most intense ions were selected for fragmentation via higher energy c-trap dissociation (resolution 15,000, AGC target 2 × 10^5^, maximum injection time 86 ms, isolation window 1.6 *m/z*, normalized collision energy 27%). Spray voltage was set to 1.9 kV, with heated capillary temperature at 285°C and funnel RF level at 45. Column temperature was set at 35°C and controlled with a Butterfly heater (Phoenix PST).

Raw data were analyzed by MaxQuant software version 2.3.1.10 (53), and the peptide list was searched against the *Homo sapiens* Uniprot protein sequence database [December 2020, only reviewed entries appended with common laboratory contaminants (cRAP database, 247 entries)] using the Andromeda search engine (54). The following settings were applied: trypsin (specificity set as C-terminal to arginine and lysine) with up to two missed cleavages, mass tolerances set to 20 ppm for the first search and 4.5 ppm for the second search. Oxidation of M, acetylation of N-termini, phosphorylation of S, T, Y, acetylation of K, and ubiquitination (GlyGly) were chosen as variable modifications, and carbamidomethylation of cysteine was chosen as static modification. FDR was set to 1% on peptide and proteins levels with a minimum length of seven amino acids and was determined by searching a reverse database. Peptide identification was performed with an allowed initial precursor mass deviation up to 7 ppm and an allowed fragment mass deviation of 20 ppm. For all other search parameters, the default settings were used. Label-free quantification (LFQ) was done using the XIC-based in-built LFQ algorithm (55) integrated into MaxQuant. Data analysis was performed in Perseus within the MaxQuant computation platform and in the *R* statistical computing environment.

Due to sample collection logistics, including age limitations on collection method, some samples used for proteomic data generation were plasma and some were sera. We observed confounding across samples by age, collection method, and sample type (Fig. S10). These data were only able to be considered cross-sectionally rather than longitudinally. In total, proteomics data were generated from samples taken at 2 months (*N* = 45). In total, 250 proteins were measured.

### Statistical analyses

For all analyses, a *P*-value <0.05 was considered significant unless otherwise stated in the results, and where appropriate FDR correction (Benjamini-Hochberg or double FDR) was applied.

For testing the relationship between antibiotic use and vaccine response, a total of 10 hypotheses were tested related to the association between antibiotic use and vaccine response. These hypotheses are not independent but organized into four groups: cumulative use, recent use, current use, and use at birth. Then within each group, there exists individual hypothesis pertaining to various time points of interest. The double FDR procedure imposes type I error control at both the group and the individual hypotheses level by leveraging the group structure during the multiplicity correction step and is therefore more appropriate than the traditional Benjamini-Hochberg (BH) procedure, which implicitly assumes the independence among hypotheses.

Confidence intervals for demographic variables were calculated in *R* using the “prop.test” function (Table S8). To test relationships between antibiotic usage and vaccine response, we assumed that vaccinations were received on the same day as the 2-month, 4-month, and 6-month well-check for each child. Analysis of antibiotic exposure was limited to drugs given systemically, with topical antibiotic exposures excluded.

To associate diversity metrics and individual feature abundances with categorical vaccine outcome, Mann-Whitney *U* tests were used. FDR correction was done using the Benjamini-Hochberg method per time point per outcome. To correlate diversity metrics and individual features with continuous vaccine outcome, Spearman’s rho was used. Enrichment analyses were done using nominally significant features and KEGG modules (56). Modules with less than five KOs were removed from the analysis and only KOs detected in the experiment were used as the total number of KOs for the analysis. Fisher’s exact test was used to calculate enrichment and was FDR corrected across all modules.

## Data Availability

Data are available in the Harvard University Dataverse. The metagenome sequencing data are available in SRA PRJNA961698. Code is available in FigShare under the DOI: 23589789.

## SUPPLEMENTAL MATERIAL

Supplemental Figures msystems.00661-23-s0001.pdf Fig. S1 to S10.

Supplemental Text msystems.00661-23-s0002.pdf Supplemental text that supports the paper.

Supplemental Tables msystems.00661-23-s0003.pdf Tables S1 to S8.

ASM does not own the copyrights to Supplemental Material that may be linked to, or accessed through, an article. The authors have granted ASM a non-exclusive, world-wide license to publish the Supplemental Material files. Please contact the corresponding author directly for reuse.
