# Supplementary material for: Very early life microbiome and metabolome correlates with primary vaccination variability in children
Source: mSystems. 2023 Aug 23;8(5):e00661-23. doi: 10.1128/msystems.00661-23 (PMC10654091; doi:10.1128/msystems.00661-23)
Supplement: Supplemental Figures — Fig. S1 to S10. [file msystems.00661-23-s0001.pdf]

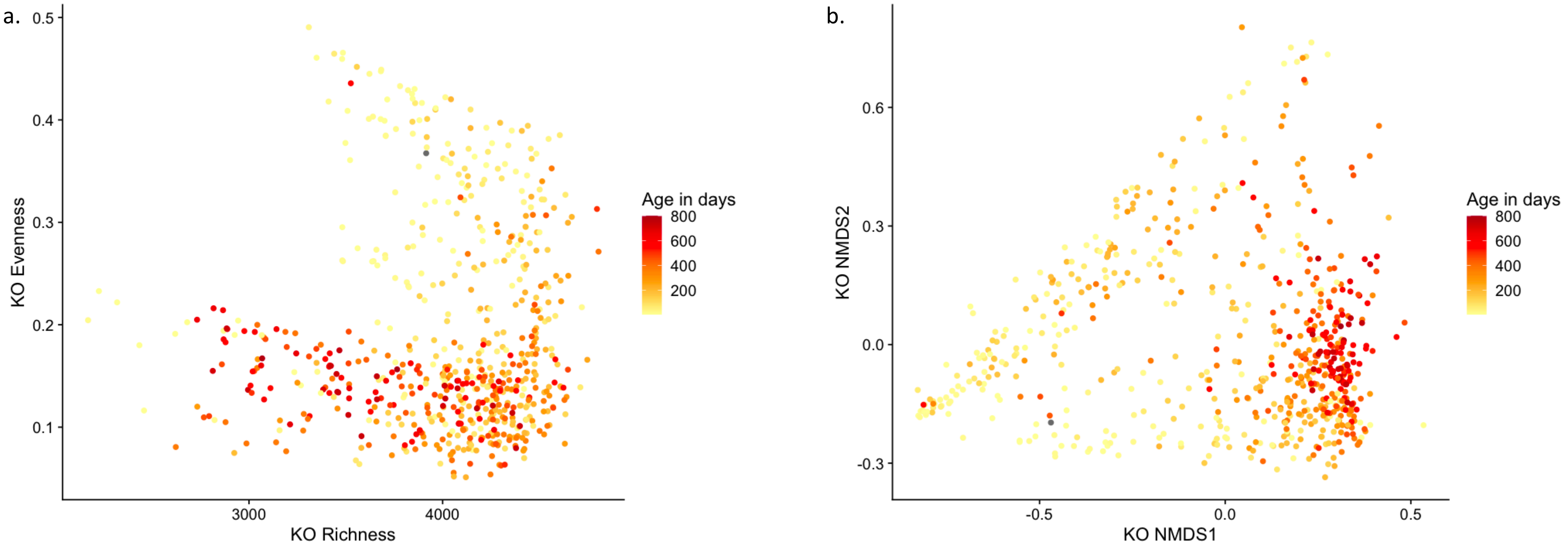

Fig. S1 Stool KO diversity shifts over time.

a) Alpha diversity showing richness (number of KOs) on x-axis and evenness (Simpson's E) on the y-axis. Shows young samples with high evenness while older samples have lower evenness and a wide spread of richness values.
b) NMDS plot showing beta diversity values for Stool KOs. Samples from later timepoints are concentrated to the right side of the plot with large amounts of spread for lower values.

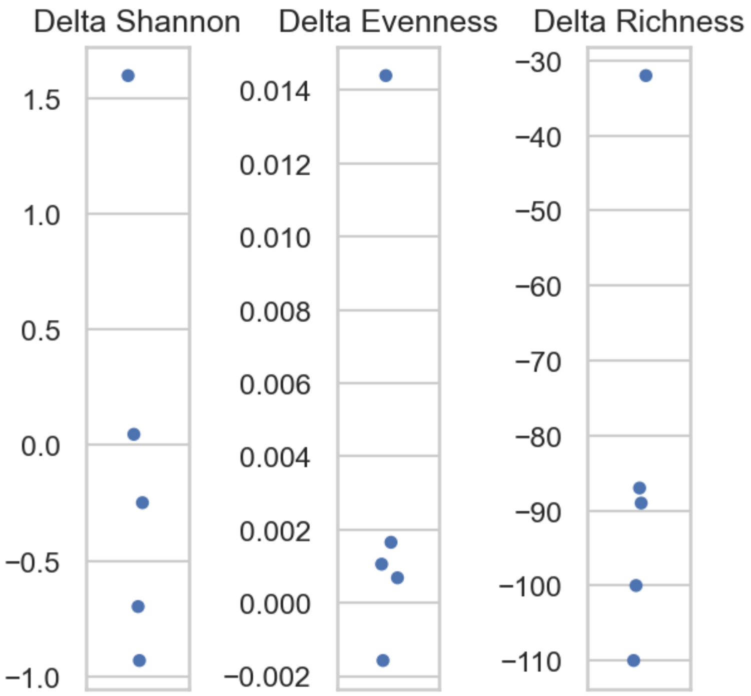

Fig. S2 Change in alpha diversity of genus level Kraken taxonomy from stool metagenomics after antibiotics usage.
We identified pairs of stool samples where one sample was within 7 days before the beginning of a course of antibiotics and one sample was within 7 days of the end of the same course of antibiotics (N=5). Shannon diversity and evenness did not show a consistent pattern across all sample pairs, but all showed a decrease in richness. Due to the sample size this decrease is not statistically significant (Wilcoxon Signed Rank Test).

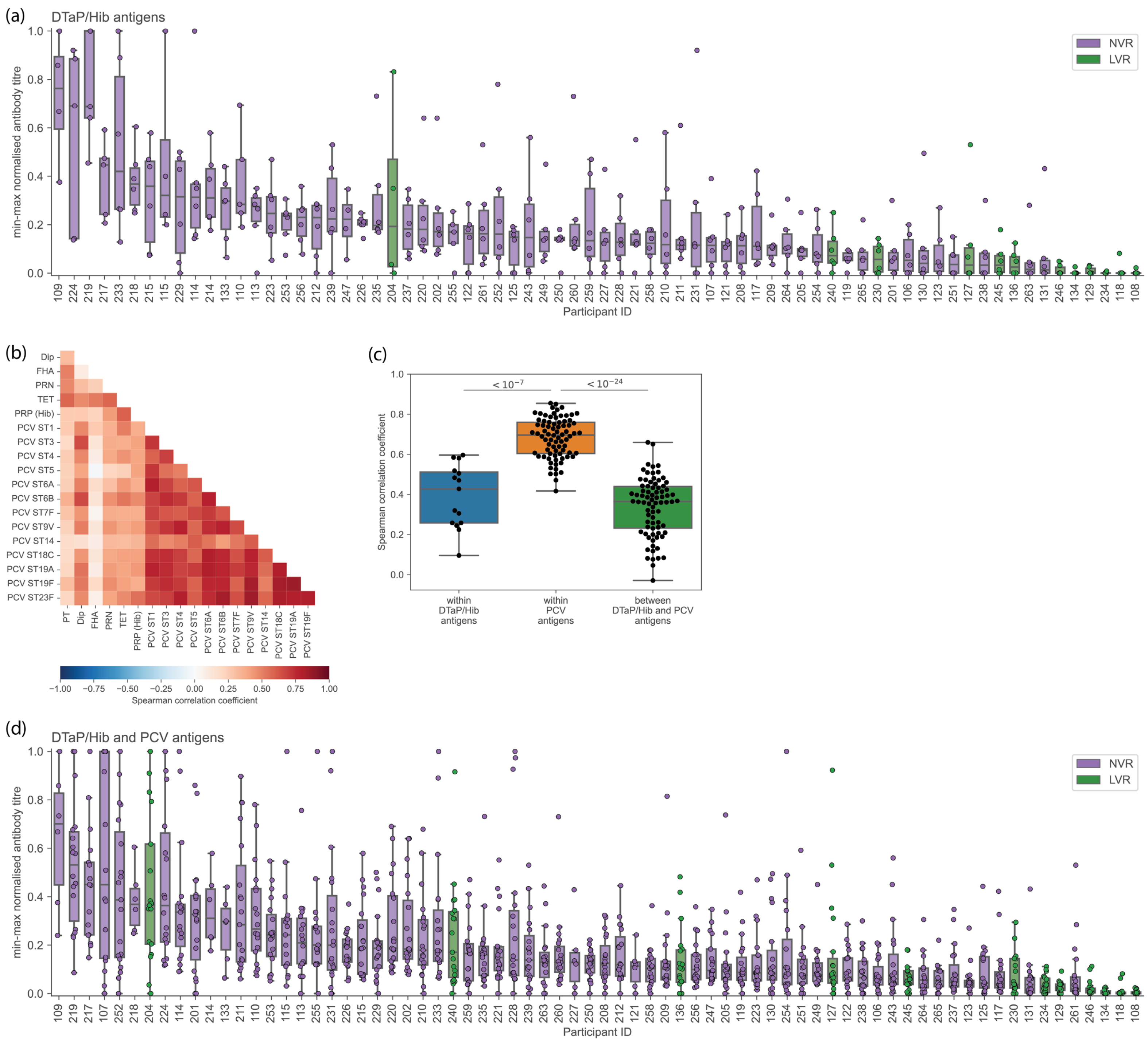

Fig. S3 Vaccine response at 1 year of age.

a) Min-max normalized antibody titer against the six DTaP/Hib antigens tested for each subject. Subjects are ordered by median value across antigens and are colored by categorical vaccine response status. NVR = normal vaccine responder, LVR = low vaccine responder.

b) Correlation between antibody titers against each of the 19 antigens tested.

c) Correlation coefficients of antibody titers between pairs of antigens, grouped by whether the pair of antigens are both from the DTaP/Hib vaccine (left, blue), both from the PCV vaccine (center, orange), or from different vaccines (right, green). P values shown are from a Mann Whitney U test. No statistical significance when testing within DTaP/Hib (blue) against between vaccines (green).

d) As for (a) but including DTaP/Hib and PCV antigens.

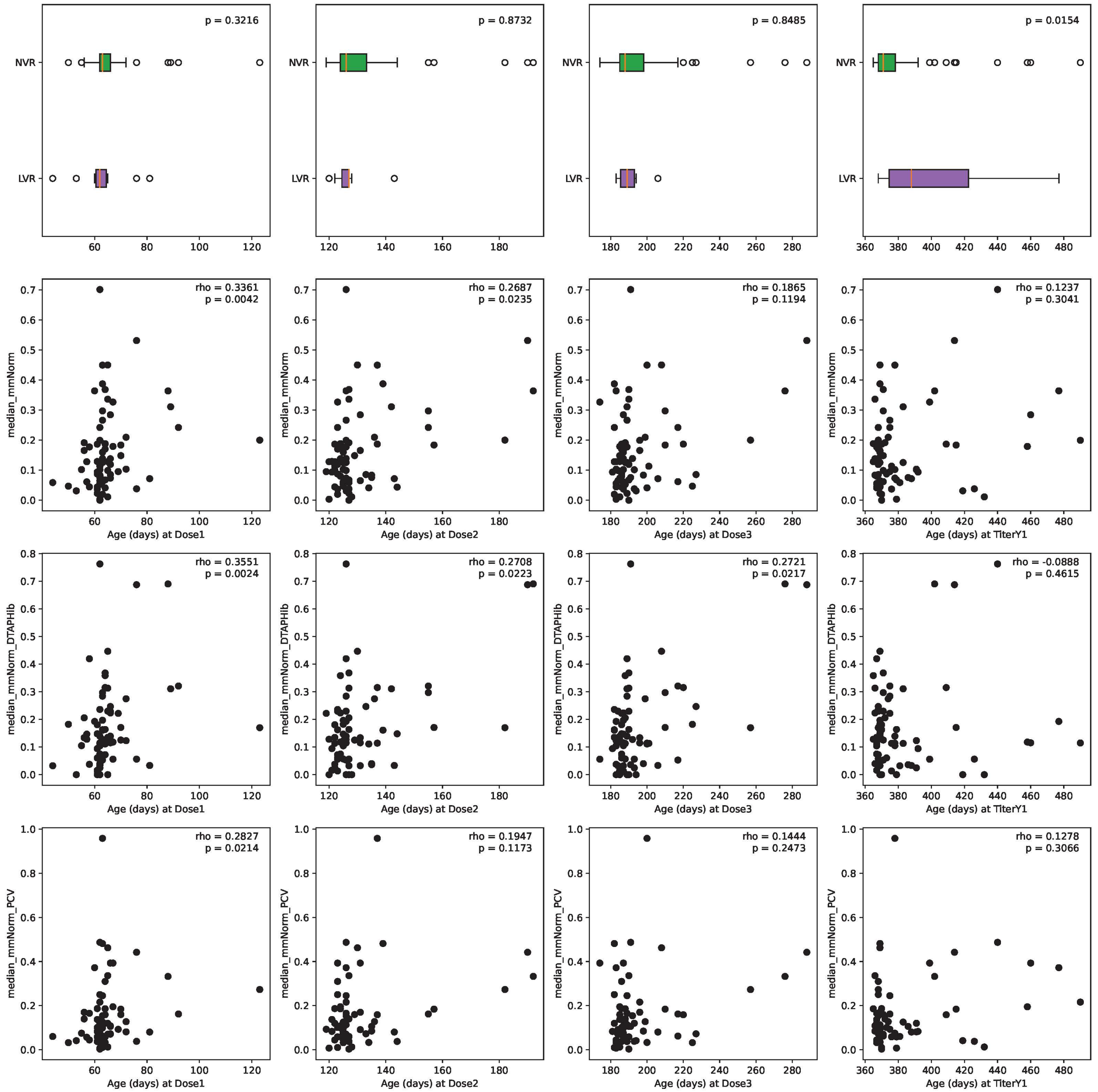

Fig. S4 Relationship between age at vaccination dose and vaccine response.

The relationship between age in days at time of dose 1 (scheduled at 2 months, left column), dose 2 (scheduled at 4 months, 2nd column from the left), dose 3 (scheduled at 6 months, 3rd column from the left), and titer measurement (1 year, right hand column) and vaccine response (rows), measured as categorical response status (top row), cross-vaccine median titer (second row), median DTaP/Hib titer (third row) and median PCV titer (bottom row). P values shown are without any multiple test correction and are from a Mann Whitney U test (top row) or a Spearman correlation (other rows).

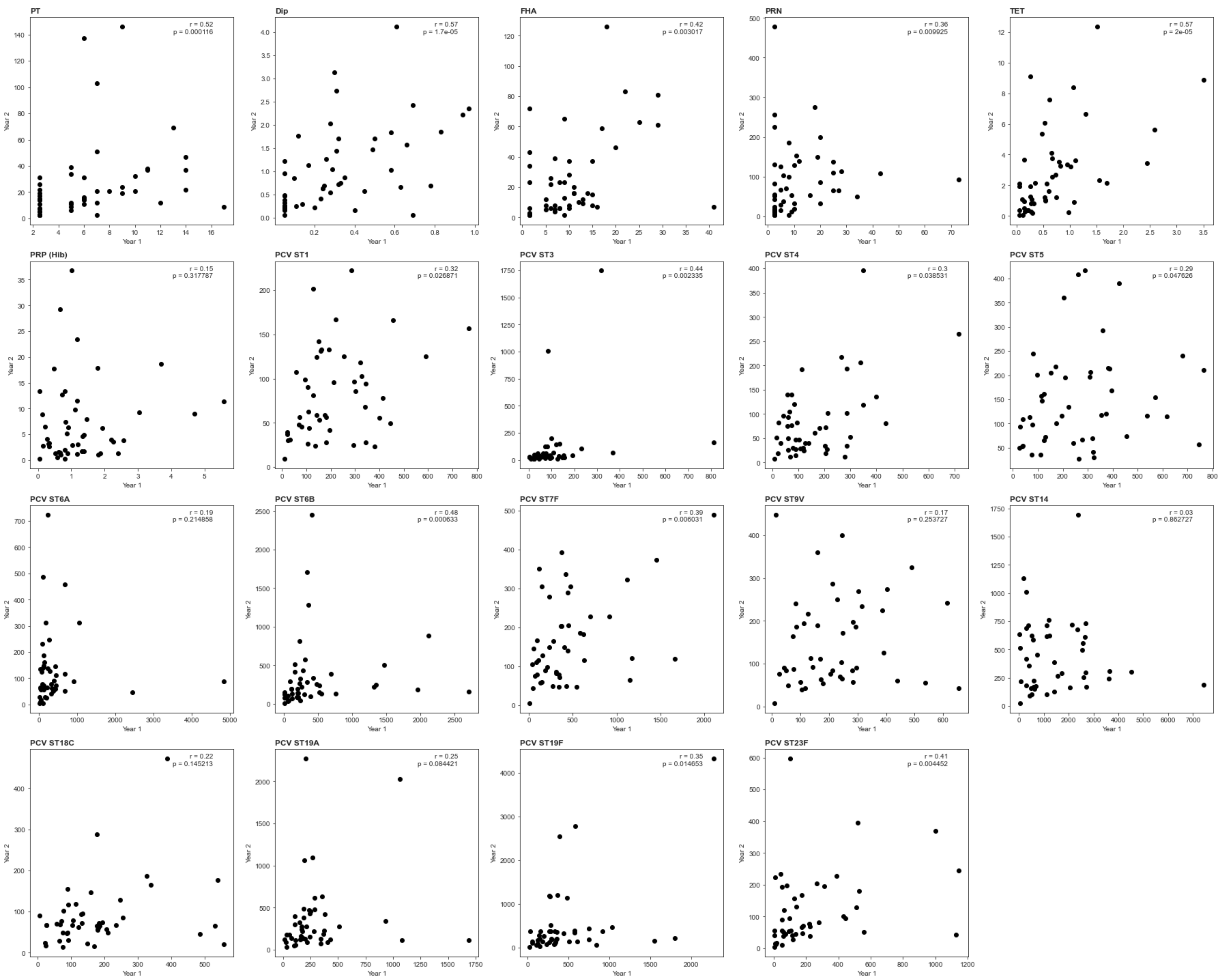

Fig. S5 Vaccine response is correlated between year 1 and year 2.
The relationship between measured antibody response at year 1 (x-axis) and year 2 (y-axis) for each of the 19 antigens in this study. Units are IU/mL for Dip and TET, ug/mL for PRP, ELU/ml for PT, PRN and FHA and ng/mL for all PCV antigens

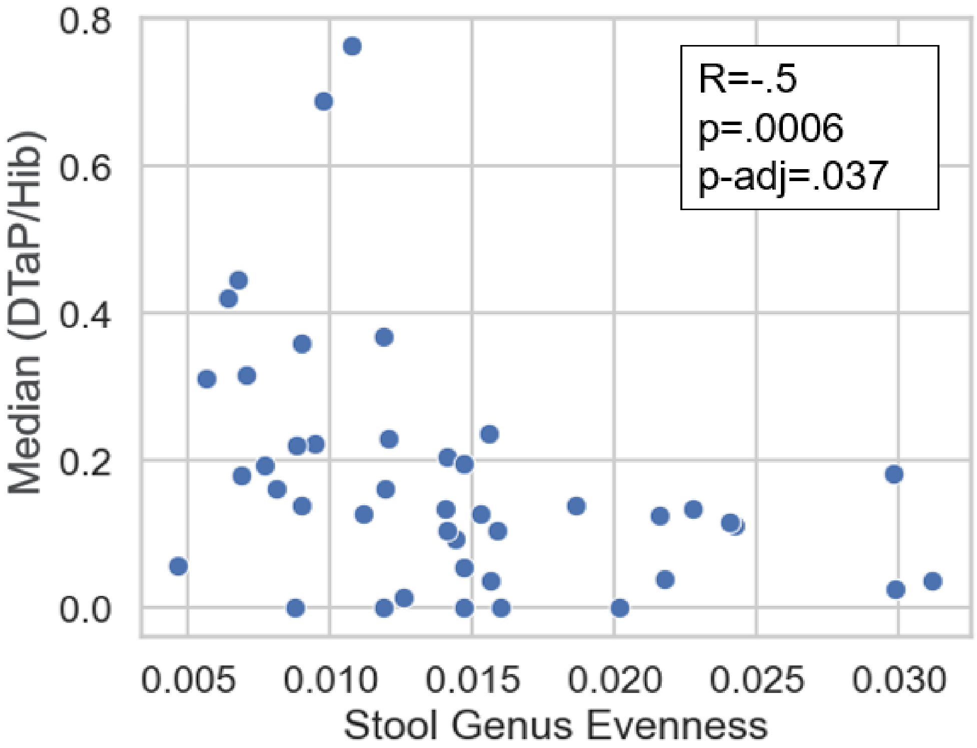

Fig. S6 Correlation between evenness at 2 months and median DTaP/Hib titer at 1 year.
Each point is a sample from 2 months of life. There is a significant negative correlation between evenness of the genuses in the samples and DTaP/Hib titer response.

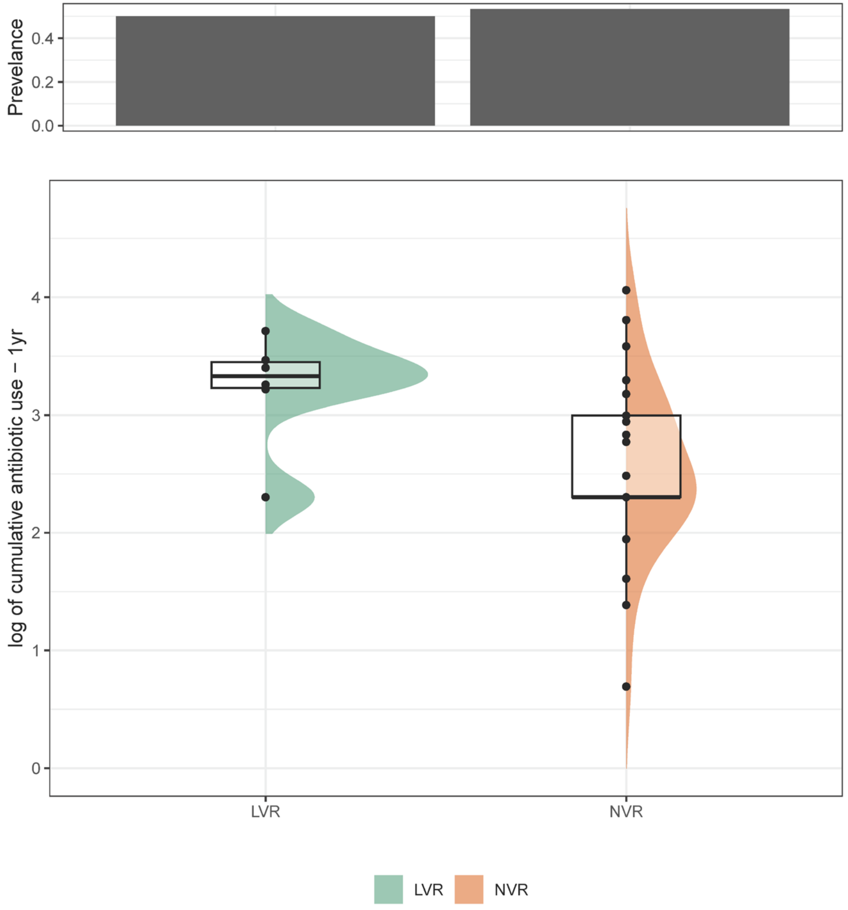

Fig. S7 Raincloud plot illustrating the association between the cumulative oral antibiotic use at 1 year, which follows a zero inflated Poisson distribution, and vaccine response. Bar plots on the top panel corresponds to the proportion of children having > 0 exposures to oral antibiotics during the 1st year. Density plots and boxplots on the bottom panel correspond to the cumulative days (in the natural log scale) of oral antibiotic exposures only among those children who were exposed to oral antibiotics

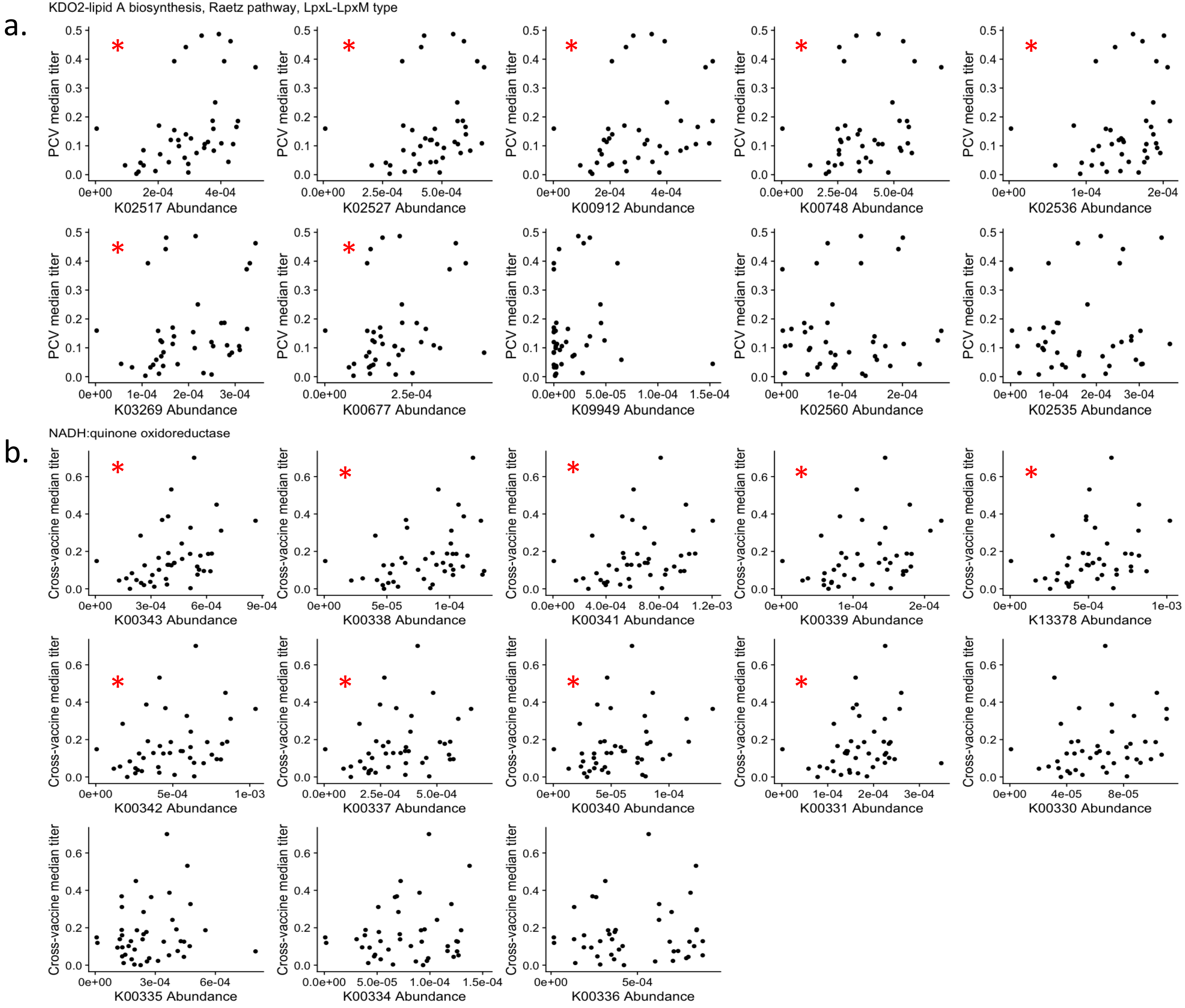

Fig. S8 Correlation between KO abundances from significant modules and median titer values at 1 year.
Each point is a sample from 2 months of life. Stars indicate correlations that were nominally significant.

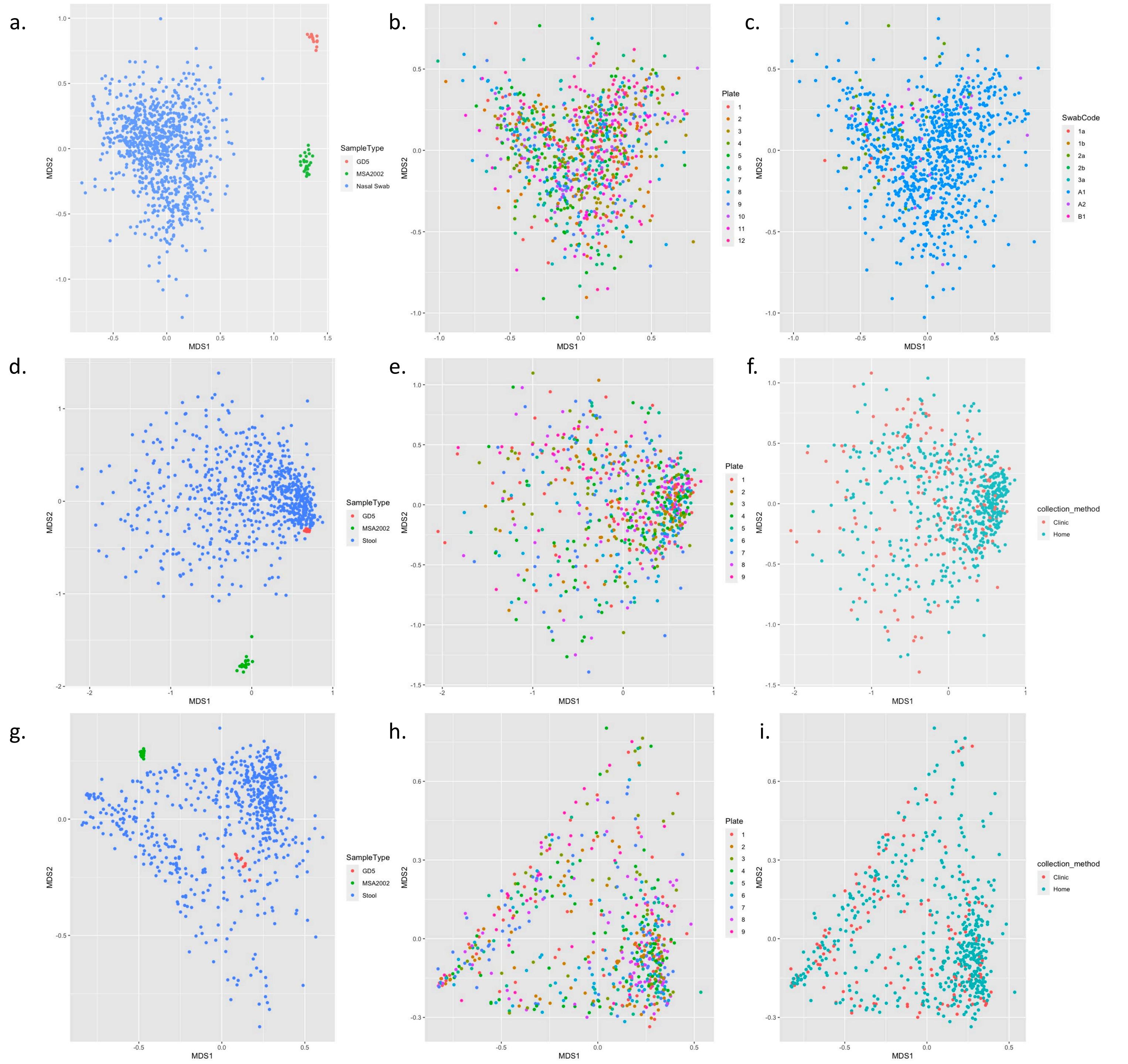

Fig. S9 Microbiome data quality control.
Plots are all NMDS plots calculated from Bray-Curtis distance. Top row shows Nasal (nasopharyngeal) 16S data (a-c), middle row shows genus level Kraken taxonomy data from stool metagenomics (d-f) and the bottom rows shows KO data from stool metagenomics (g-i). The first column includes subject samples and control samples and shows clear clustering on sample data away from controls across data sets (a, d, g). The middle column shows subject samples colored by sequencing plate and shows no clustering (b, e, h). The last column shows subject samples colored by collection method. In the nasopharynx non-A1 swaps are clustered toward the top left of the plot which is also were samples from young subjects are located (c). This is because other swab types are used on young children. When isolating samples with different swab types at the same age there is no clustering. In the stool metagenomics derived data types there is no clustering based on whether samples were collected at home or in the clinic (f, i).

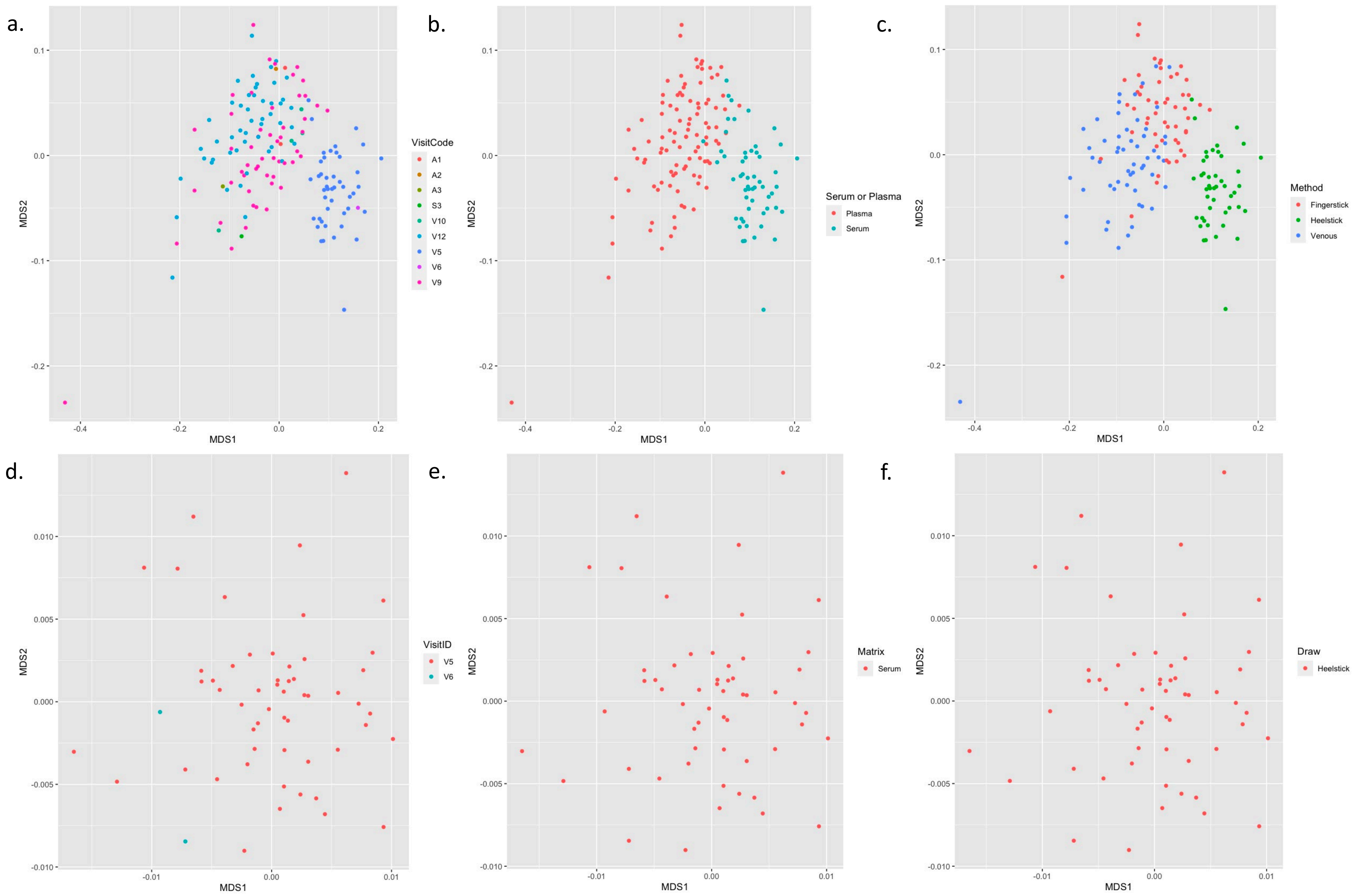

Fig. S10 Metabolome and proteome QC. Plots are all NMDS plots calculated from Bray-Curtis distance. The top row are from metabolomics (a-c) and the bottom row are from proteomics data (d-f). The first column is samples colored by the visit code for each sample (a, d). The second column is colored by whether an analysis was done on data from the blood plasma or serum (b, e). The third column shows subject samples colored by the method of blood collection (c, f). The metabolomics shows clear separation of samples based on both serum or plasma usage as well as blood collection method. As a result of this confounding analysis for metabolomics and proteomics was done cross-sectionally.
