# Supplementary material for: Very early life microbiome and metabolome correlates with primary vaccination variability in children
Source: mSystems. 2023 Aug 23;8(5):e00661-23. doi: 10.1128/msystems.00661-23 (PMC10654091; doi:10.1128/msystems.00661-23)
Supplement: Supplemental Text — that supports the paper. [file msystems.00661-23-s0002.pdf]

Supplemental Text

Densely sampled subject demonstrates generalizable developmental trajectory. The dense sampling from subject 108 in this cohort allowed us to study in detail the progression of the stool and nasal microbiomes over the first two years of life, providing many examples of common development profiles that could be generalized across the study population. Subject 108 provided 29 stool samples along with 18 nasal samples (Fig. 2c). This subject visited the clinic to be treated for AOM 6 times and received 12 courses of antibiotics in total.

Subject 108's NP samples from the first 75 days of life were dominated by *Staphylococcus* (99% in both samples), but later time points were much more diverse. Similarly, across the entire cohort, *Staphylococcus* was the most abundant genus in 100 of 240 samples (42%) from before day 75 and 37 of 704 samples (5%) from after day 75. The samples from Subject 108 at 190 and 258 days represented the transition to the types of communities that were common across the rest of the time series. The remaining NP samples were made up of a plurality of members of the *Moraxella* (order *Pseudomonadales*) or *Haemophilus* (order *Pasteurellales*) genus with *Lactobacillales* being the only other major member of the community. Across the cohort, Moraxella was the most abundant taxa in 417 of 704 samples older than 75 days.

Haemophilus spp. have been associated with AOM status which is often treated with antibiotics. In this subject, *Haemophilus* made up the plurality of the NP community in 4 out of 5 samples where the subject was visiting the clinic because of AOM. In the rest of the cohort, 30% of samples at the time of a clinic visit for AOM had *Haemophilus* as the most abundant genus compared to only 6% of other samples.

Similarly, frequent sampling of Subject 108 allowed us to observe clear development of the stool microbiome (Fig. 2c). Before transition to solid food at around 10 months, the subject's diet was primarily breast milk. *Bifidobacteriales* were at least 20% of the community present in 7 out of 8 pre-weaning samples from subject 108. Across the cohort, samples from before day 300 of life, the approximate time of weaning, 16% of samples had *Bifidobacteriales* abundances of >40% while after day 300 this dropped to <1% of samples. In subject 108, the stool microbiome rapidly shifted between birth and 10 months until the sample taken at day 301 when the subject had started solid food and the diet was <50% breast milk, meaning that the subjects' diet was made up of less than 50% breast milk. This established a more consistent norm through the rest of the study. After day 300, all but one sample from subject 108 was dominated by *Bacteroidales* with some *Clostridales*. This was mirrored across the cohort where on average *Bacteroides* makes up 59% of the gut microbiome in samples collected after day 300.

On day 456, subject 108 began taking antibiotics after a visit for AOM. The sample from that day showed a profile in line with the rest of their post-weaning samples. On day 459, 3 days after they started on antibiotics, their gut microbiome decreased in alpha diversity and had a bloom of *Enterobacter* that was not present three days earlier. Looking across the cohort there were only a small subset of participants for whom we had recent stool samples before and after oral antibiotic treatment (n = 5, within 7 days). In those samples we observed a median reduction in number of observed species of 60 (median 371 species before antibiotics to median 291 species after antibiotic) but the sample size was insufficient to reach statistical significance (Fig. S2). It also is not clear if the *Enterobacter* bloom was a result of taking antibiotics as similar blooms do not occur in other samples from the subject where antibiotics were taken recently

(samples from days 373, 470, 679, 702). Additionally, this level of *Enterobacter* was not seen in any other post-antibiotics, post-weaning samples in this cohort; the maximum *Enterobacter* abundance in post-antibiotic post-weaning samples was 5%. As such, this feature of subject 108 was likely to be unique and not generalizable.

The dense sampling from subject 108 in this cohort allowed us to study in detail the progression of the stool and nasal microbiomes over the first two years of life, providing many examples of common development profiles that could be generalized across the study population.
