# Supplementary material for: Very early life microbiome and metabolome correlates with primary vaccination variability in children
Source: mSystems. 2023 Aug 23;8(5):e00661-23. doi: 10.1128/msystems.00661-23 (PMC10654091; doi:10.1128/msystems.00661-23)
Supplement: Supplemental Tables — Tables S1 to S8. [file msystems.00661-23-s0003.pdf]

Table S1. Cohort metadata.

PrimaryKey	Subject ID	NVR/LVR	Date of Birth	Gender	Birth Height (cm)	Birth Height Percentile	Birth Weight (kg)	Birth Weight Percentile	Birth Head Cir. (cm)	Birth Head Cir. Percentile
Baby101	101	None Recorded	1/24/2018	Male	Not Documented	Not Documented	3.646	Not Documented	Not Documented	Not Documented
Baby102	102	None Recorded	2/20/2018	Male	50	50	3.35	37	34.5	25
Baby103	103	None Recorded	2/21/2018	Female	52	84	3.41	51	36.5	85
Baby104	104	None Recorded	3/12/2018	Female	55.2	98	3.615	61	35.6	64
Baby105	105	None Recorded	3/30/2018	Female	50.8	72.31	3.97	89.09	34	32.73
Baby106	106	NVR	3/27/2018	Male	51	Not Documented	3.08	Not Documented	35	Not Documented
Baby107	107	NVR	4/2/2018	Male	52.5	83	3.65	59	35	34
Baby108	108	LVR	3/28/2018	Male	53.3	89.86	3.82	71.12	34.5	25.47
Baby109	109	NVR	4/17/2018	Male	Not Documented	Not Documented	Not Documented	Not Documented	Not Documented	Not Documented
Baby110	110	NVR	5/7/2018	Female	53	92	3.195	34	33	13
Baby111	111	None Recorded	5/5/2018	Female	51	65	3.33	36	35	34
Baby112	112	None Recorded	5/3/2018	Male	51.4	71	3.41	41	34.5	25
Baby113	113	NVR	5/15/2018	Male	54.5	96	4.03	84	36.5	65
Baby114	114	NVR	5/21/2018	Female	52.1	86	3.235	37	35	57
Baby115	115	NVR	5/24/2018	Male	48.3	26	2.97	17	32	4
Baby116	116	None Recorded	5/26/2018	Male	Not Documented	Not Documented	2.872	13	Not Documented	Not Documented
Baby117	117	NVR	6/10/2018	Male	49.5	42	3.355	51	33.5	22
Baby118	118	LVR	6/25/2018	Male	50.8	62	3.71	63	33.5	13
Baby119	119	NVR	6/30/2018	Female	50.5	68	3.565	64	35	57
Baby120	120	None Recorded	6/30/2018	Female	50.8	72.31	2.775	10.93	35	57
Baby121	121	NVR	6/30/2018	Male	52.1	78	4.025	83	35.5	43
Baby122	122	NVR	7/5/2018	Female	51	75	3.655	71	34	33
Baby123	123	NVR	7/8/2018	Male	49.5	43	2.94	15	35	34
Baby124	124	None Recorded	8/22/2018	Male	Not Documented	Not Documented	3.952	79.44	Not Documented	Not Documented
Baby125	125	NVR	8/16/2018	Male	50.8	68.4	3.623	70.9	34.9	63.3
Baby126	126	None Recorded	8/27/2018	Male	Not Documented	Not Documented	Not Documented	Not Documented	Not Documented	Not Documented
Baby127	127	LVR	8/31/2018	Female	Not Documented	Not Documented	Not Documented	Not Documented	Not Documented	Not Documented
Baby128	128	None Recorded	8/26/2018	Male	52.5	82.94	3.47	45.58	35	33.79
Baby129	129	LVR	9/5/2018	Female	49	45.3	3.415	51.3	34	32.7
Baby130	130	NVR	10/27/2018	Female	Not Documented	Not Documented	Not Documented	Not Documented	Not Documented	Not Documented
Baby131	131	NVR	11/8/2018	Male	50.8	62.03	3.91	76.94	37.5	83.61
Baby132	132	None Recorded	11/18/2018	Female	53.3	93.32	3.43	52.58	35.6	69.39
Baby133	133	NVR	11/19/2018	Female	55.9	98.96	4.043	91.84	Not Documented	Not Documented
Baby134	134	LVR	11/22/2018	Male	53.3	89.86	3.905	76.62	36.5	64.78
Baby135	135	None Recorded	11/26/2018	Female	48.5	37.26	3.21	34.98	34	32.73
Baby136	136	LVR	12/14/2018	Female	49.5	53.42	3.275	39.95	34.5	44.81
Baby201	201	NVR	2/22/2018	Female	47.76	20	2.911	23	33	23
Baby202	202	NVR	2/27/2018	Male	49.53	43	3.079	29	Not Documented	Not Documented
Baby203	203	None Recorded	2/28/2018	Female	52.5	96	3.246	52	32.5	12
Baby204	204	LVR	2/26/2018	Female	45.7	Not Documented	2.605	Not Documented	30.5	Not Documented
Baby205	205	NVR	2/28/2018	Male	53.34	97	3.572	67	35.5	79
Baby206	206	None Recorded	3/9/2018	Female	49.5	51	3.461	64	33.7	39
Baby207	207	None Recorded	3/11/2018	Female	Not Documented	Not Documented	4.196	Not Documented	Not Documented	Not Documented
Baby208	208	NVR	3/7/2018	Female	48.26	Not Documented	2.58	Not Documented	33.5	Not Documented
Baby209	209	NVR	3/14/2018	Female	Not Documented	Not Documented	3.175	44	32	6
Baby210	210	NVR	3/10/2018	Female	52.07	94	2.821	17	35	83
Baby211	211	NVR	3/11/2018	Male	55.88	99	3.734	83	Not Documented	Not Documented
Baby212	212	NVR	3/18/2018	Male	50.8	Not Documented	3.969	89	Not Documented	Not Documented
Baby213	213	None Recorded	3/31/2018	Female	47.6	20.3	2.747	13.3	34.9	80.5
Baby214	214	NVR	4/4/2018	Female	48.26	31.6	2.611	7.4	Not Documented	Not Documented
Baby215	215	NVR	3/29/2018	Male	53.34	96.6	3.909	86.4	Not Documented	Not Documented
Baby216	216	None Recorded	4/6/2018	Male	49.53	42.25	3.303	46	34.5	51.2
Baby217	217	NVR	4/8/2018	Female	51.43	89.1	4.006	94.4	Not Documented	Not Documented
Baby218	218	NVR	4/3/2018	Male	52.1	87.9	3.561	66.6	33.7	27.4
Baby219	219	NVR	4/10/2018	Male	51.43	79.4	3.926	87.1	Not Documented	Not Documented
Baby220	220	NVR	4/13/2018	Female	49.78	63.3	3.08	36.7	32	5.6
Baby221	221	NVR	4/16/2018	Female	50.8	81.3	3.6	69.5	33	23
Baby222	222	None Recorded	4/17/2018	Male	52.1	87.9	4.054	91.1	34.3	44.8
Baby223	223	NVR	4/27/2018	Female	48.26	19.5	3.084	36.7	Not Documented	Not Documented
Baby224	224	NVR	4/22/2018	Female	50.8	81.3	3.025	32.6	33.7	44
Baby225	225	None Recorded	4/30/2018	Male	Not Documented	Not Documented	4.425	97.8	35.6	81.6
Baby226	226	NVR	5/3/2018	Female	53.5	99	4.278	98	Not Documented	Not Documented
Baby227	227	NVR	4/30/2018	Female	52.7	97.2	3.595	78.2	Not Documented	Not Documented
Baby228	228	NVR	5/7/2018	Male	50.8	68.4	3.087	29.5	Not Documented	Not Documented
Baby229	229	NVR	4/29/2018	Male	48.3	20	3.161	35	34.9	63
Baby230	230	LVR	5/10/2018	Male	Not Documented	Not Documented	2.353	1	Not Documented	Not Documented
Baby231	231	NVR	5/9/2018	Female	48.3	32.3	3.206	48	31.5	2.2
Baby232	232	None Recorded	5/14/2018	Female	47	12.5	2.43	2.8	31.8	3.9
Baby233	233	NVR	5/21/2018	Female	49.5	54	3.615	67.5	34.9	55
Baby234	234	LVR	6/15/2018	Male	53.3	90	4.508	98	36.5	57
Baby235	235	NVR	6/13/2018	Male	56.5	99	4.536	98	36	42
Baby236	236	None Recorded	6/12/2018	Female	48.3	33.51	3.657	70.68	Not Documented	Not Documented
Baby237	237	NVR	6/20/2018	Female	53.3	93	4.285	97	36.2	81
Baby238	238	NVR	6/20/2018	Female	50.8	79.31	3.35	45.97	34.3	39.64
Baby239	239	NVR	6/24/2018	Male	50.8	62.03	3.5	47.77	33	9.41
Baby240	240	LVR	6/21/2018	Male	48	23	2.719	9	33	9
Baby241	241	None Recorded	6/24/2018	Male	53.3	96.6	3.975	88.78	35.6	80.64
Baby242	242	None Recorded	6/29/2018	Male	Not Documented	Not Documented	3.09	29.53	Not Documented	Not Documented
Baby243	243	NVR	7/11/2018	Female	50.8	72.31	3.155	30.99	32.4	6.08
Baby244	244	None Recorded	7/15/2018	Male	52.1	78.47	3.487	46.82	Not Documented	Not Documented
Baby245	245	LVR	7/22/2018	Male	49.5	43.15	3.955	79.61	36.8	71.6
Baby246	246	LVR	7/28/2018	Male	47	13.21	2.51	4.76	33	9.41
Baby247	247	NVR	8/24/2018	Male	50.8	62.03	3.639	58.11	Not Documented	Not Documented
Baby248	248	None Recorded	8/19/2018	Female	48.9	43.64	2.765	10.59	31.8	2.19
Baby249	249	NVR	8/24/2018	Male	52.1	87.59	4.23	95.28	38.1	99.79
Baby250	250	NVR	9/5/2018	Male	50.8	62.03	3.4	40.58	35.6	44.66
Baby251	251	NVR	8/29/2018	Male	50.8	62.03	3.66	59.67	34.9	32.43
Baby252	252	NVR	9/6/2018	Male	57	22.44	4.791	99.63	38	90.31
Baby253	253	NVR	9/27/2018	Female	54.7	97.51	4.537	99.46	36	77.59
Baby254	254	NVR	9/28/2018	Male	45.7	5.6	2.404	3.47	Not Documented	Not Documented
Baby255	255	NVR	9/27/2018	Female	Not Documented	Not Documented	2.818	Not Documented	Not Documented	Not Documented
Baby256	256	NVR	10/2/2018	Male	48.3	25.85	3.21	28.33	Not Documented	Not Documented
Baby257	257	None Recorded	10/12/2018	Male	54.6	99.37	3.537	64.91	33	12.48
Baby258	258	NVR	10/23/2018	Male	50.5	67.76	3.745	78.41	Not Documented	Not Documented
Baby259	259	NVR	10/26/2018	Female	50.8	81.25	2.973	28.08	32	5.62
Baby260	260	NVR	11/5/2018	Male	53.3	85.73	4.076	85.57	Not Documented	Not Documented
Baby261	261	NVR	11/1/2018	Male	57.2	99.99	3.75	78.69	35.5	67.99
Baby262	262	None Recorded	11/22/2018	Female	Not Documented	Not Documented	Not Documented	Not Documented	Not Documented	Not Documented
Baby263	263	NVR	11/24/2018	Female	52.7	86.9	3.912	86.48	36.3	77.58
Baby264	264	NVR	12/4/2018	Male	55.9	99.86	4.138	93.44	36.5	92.74
Baby265	265	NVR	12/6/2018	Male	48.3	11.8	3.341	49.56	33	7.32

PrimaryKey	Subject ID	Race #1	Race #2	Gestation	Delivery Type	Age of Mother	Breastfed at 6 months	Breastfed at enrollment	Daycare at 6 months	Complications During Pregnancy?
Baby101	101	White/Caucasian		39w	Vaginal	29	Not Documented	<50%	Not Documented	Yes
Baby102	102	Arab/North African		39w 6d	Vaginal	28	Not Documented	1	Not Documented	No

Baby103	103	White/Caucasian		41w 3d	Vaginal	31	1	1	Yes	No
Baby104	104	White/Caucasian		40w	Vaginal	33	Not Documented	1	Not Documented	No
Baby105	105	White/Caucasian		39w	Vaginal	41	0-5%	0-5%	No	Yes
Baby106	106	White/Caucasian		40w	Vaginal	Not Documented	Greater 50%	1	Yes	No
Baby107	107	White/Caucasian		39w 1d	C-Section	30	1	<50%	Yes	No
Baby108	108	White/Caucasian		41w	C-Section	32	1	>50%	No	No
Baby109	109	Black		40w	Vaginal	19	0-5%	>50%	No	No
Baby110	110	White/Caucasian	Hispanic	40w	Vaginal	29	0-5%	1	No	No
Baby111	111	White/Caucasian		40w	Vaginal	30	1	1	No	No
Baby112	112	White/Caucasian		39w 1d	C-Section	38	1	1	No	No
Baby113	113	White/Caucasian		40w 5d	Vaginal	31	0-5%	1	No	No
Baby114	114	White/Caucasian	Hispanic	39w	C-Section	39	1	1	No	No
Baby115	115	Black		39w	Vaginal	29	0-5%	0-5%	No	No
Baby116	116	White/Caucasian		40w 3d	Vaginal	33	0-5%	1	Yes	No
Baby117	117	Black		39w 3d	Vaginal	24	Greater 50%	1	No	No
Baby118	118	Black	White/Caucasian	39w 4d	Vaginal	28	Greater 50%	1	No	No
Baby119	119	White/Caucasian		40w 3d	Vaginal	28	1	1	No	No
Baby120	120	White/Caucasian		40w 3d	Vaginal	28	Not Documented	1	Not Documented	No
Baby121	121	White/Caucasian		40w	Vaginal	31	1	1	No	No
Baby122	122	White/Caucasian		40w 6d	Vaginal	37	0-5%	1	Yes	No
Baby123	123	White/Caucasian		41w 1d	C-Section	37	0-5%	1	No	No
Baby124	124	Black	White/Caucasian	37w	Vaginal	27	0-5%	<50%	No	Yes
Baby125	125	Hispanic		39w 5d	C-Section	29	1	1	No	No
Baby126	126	White/Caucasian		41w 2d	Vaginal	32	1	1	No	No
Baby127	127	Hispanic		40w 4d	Vaginal	27	Greater 50%	>50%	No	Yes
Baby128	128	White/Caucasian		39w 4d	Vaginal	39	0-5%	0-5%	Yes	No
Baby129	129	White/Caucasian		39w	C-Section	33	Greater 50%	1	No	No
Baby130	130	White/Caucasian		38w 3d	Vaginal	32	1	1	No	Yes
Baby131	131	White/Caucasian		39w 3d	Vaginal	31	0-5%	0-5%	Yes	No
Baby132	132	Black		39w 4d	Vaginal	23	Not Documented	1	Not Documented	No
Baby133	133	White/Caucasian		39w	C-Section	33	0-5%	>50%	No	No
Baby134	134	White/Caucasian		41w 1d	Vaginal	32	1	1	No	No
Baby135	135	White/Caucasian		40w	Vaginal	23	Greater 50%	1	No	No
Baby136	136	White/Caucasian		40w 5d	Vaginal	32	0-5%	1	No	Yes
Baby201	201	White/Caucasian		37w	Vaginal	24	Not Documented	1	Not Documented	Yes
Baby202	202	Hispanic		40w	Vaginal	28	0-5%	0-5%	Yes	No
Baby203	203	White/Caucasian		37w 4d	C-Section	25	0-5%	>50%	No	Yes
Baby204	204	White/Caucasian		39w 1d	Vaginal	37	Lesser 50%	>50%	Yes	No
Baby205	205	White/Caucasian		39w	Vaginal	34	1	1	No	Yes
Baby206	206	White/Caucasian	Hispanic	40w	Vaginal	31	0-5%	0-5%	No	No
Baby207	207	White/Caucasian		39w	Vaginal	24	0-5%	0-5%	No	No
Baby208	208	White/Caucasian		Not Documented	C-Section	43	0-5%	1	No	Yes
Baby209	209	Asian		40w	Vaginal	29	0-5%	>50%	No	No
Baby210	210	White/Caucasian		37w 1d	C-section	Not Documented	0-5%	0-5%	No	Yes
Baby211	211	White/Caucasian		41w	Vaginal	30	1	1	No	No
Baby212	212	Not Documented		39w 6d	Vaginal	23	1	1	No	No
Baby213	213	Black	White/Caucasian	39w 6d	Vaginal	28	Not Documented	0-5%	Not Documented	No
Baby214	214	White/Caucasian		39w 3d	Vaginal	23	0-5%	0-5%	No	No
Baby215	215	White/Caucasian		39w 6d	Vaginal	29	Lesser 50%	<50%	No	No
Baby216	216	Black	White/Caucasian	39w 5d	Vaginal	23	1	1	No	No
Baby217	217	White/Caucasian		40w 1d	Vaginal	22	0-5%	1	No	No
Baby218	218	Black		39w 5d	Vaginal	32	0-5%	0-5%	No	No
Baby219	219	White/Caucasian		39w 4d	C-Section	34	0-5%	>50%	No	Yes
Baby220	220	White/Caucasian		39w 1d	Vaginal	27	0-5%	>50%	No	Yes
Baby221	221	White/Caucasian		40w 5d	Vaginal	30	Greater 50%	1	No	No
Baby222	222	Black	Hispanic	38w 3d	Vaginal	22	0-5%	1	Yes	Yes
Baby223	223	White/Caucasian		40w	C-Section	28	Not Documented	0-5%	Not Documented	No
Baby224	224	Black		39w 1d	Vaginal	27	0-5%	>50%	No	No
Baby225	225	Hispanic		40w 6d	Vaginal	28	0-5%	<50%	No	Yes
Baby226	226	White/Caucasian		41w 2d	Vaginal	37	Lesser 50%	1	Yes	Yes
Baby227	227	White/Caucasian		39w	Vaginal	33	1	1	Yes	No
Baby228	228	White/Caucasian		39w	Vaginal	31	Greater 50%	1	Yes	No
Baby229	229	Hispanic		39w 4d	Vaginal	31	0-5%	1	No	No
Baby230	230	White/Caucasian		38w 4d	Vaginal	18	0-5%	<50%	No	No
Baby231	231	White/Caucasian		40w 2d	Vaginal	20	0-5%	0-5%	No	Yes
Baby232	232	Black		37w 5d	Vaginal	25	0-5%	0-5%	No	No
Baby233	233	White/Caucasian		40w 5d	Vaginal	30	0-5%	1	No	No
Baby234	234	White/Caucasian		41w	Vaginal	34	0-5%	1	Yes	No
Baby235	235	White/Caucasian		38w	C-Section	33	Greater 50%	>50%	No	No
Baby236	236	White/Caucasian		39w	C-Section	35	1	1	Yes	No
Baby237	237	White/Caucasian		41w 1d	Vaginal	31	1	1	No	No
Baby238	238	White/Caucasian		38w 2d	Vaginal	39	1	1	No	Yes
Baby239	239	White/Caucasian	Hispanic	38w 4d	Vaginal	40	0-5%	>50%	Yes	Yes
Baby240	240	Black		36w	Vaginal	26	Greater 50%	>50%	No	No
Baby241	241	White/Caucasian		38w	Vaginal	24	0-5%	>50%	No	No
Baby242	242	White/Caucasian		41w	C-Section	25	Not Documented	1	Not Documented	No
Baby243	243	White/Caucasian	Hispanic	40w 4d	Vaginal	29	Not Documented	1	Not Documented	Yes
Baby244	244	White/Caucasian		40w 1d	Vaginal	26	Not Documented	<50%	Not Documented	No
Baby245	245	White/Caucasian		39w 4d	Vaginal	31	0-5%	<50%	Yes	No
Baby246	246	White/Caucasian		37w 6d	Vaginal	30	0-5%	1	No	Yes
Baby247	247	White/Caucasian		41w 3d	Vaginal	33	0-5%	1	No	No
Baby248	248	Black		40w	Vaginal	29	Lesser 50%	1	No	No
Baby249	249	White/Caucasian		39w	C-Section	30	0-5%	>50%	No	No
Baby250	250	White/Caucasian		40w	C-Section	28	0-5%	1	No	Yes
Baby251	251	White/Caucasian		39w 3d	Vaginal	26	Greater 50%	1	Yes	Yes
Baby252	252	White/Caucasian		39w 3d	C-Section	32	Greater 50%	1	Yes	Yes
Baby253	253	White/Caucasian		39w 4d	Vaginal	28	0-5%	0-5%	No	Yes
Baby254	254	White/Caucasian		36w 4d	Vaginal	24	0-5%	1	No	No
Baby255	255	White/Caucasian		37w 6d	Vaginal	25	1	1	No	Yes
Baby256	256	White/Caucasian	Hispanic	38w 5d	Vaginal	19	0-5%	>50%	No	No
Baby257	257	White/Caucasian		38w 3d	Vaginal	20	1	1	No	No
Baby258	258	White/Caucasian	Hispanic	39w	Vaginal	24	0-5%	0-5%	No	No
Baby259	259	White/Caucasian		39w	Vaginal	29	0-5%	1	No	No
Baby260	260	White/Caucasian		40w	Vaginal	30	1	1	Yes	No
Baby261	261	White/Caucasian		39w	Vaginal	23	Greater 50%	Not Documented	No	No
Baby262	262	White/Caucasian		38w	Vaginal	35	Not Documented	1	Not Documented	No
Baby263	263	White/Caucasian		41w	Vaginal	23	Greater 50%	1	No	No
Baby264	264	White/Caucasian		42w	Vaginal	28	1	1	No	No
Baby265	265	White/Caucasian		40w 6d	Vaginal	30	0-5%	1	No	No

PrimaryKey	Subject ID	Pregnancy complication #1	Pregnancy complication #2	GBS Test Result	Flu Vac while pregnant?	Complications during labor?	Labor complication #1	Labor complication #2	Has the mother received antibiotics during pregnancy or labor?	Mother Antibiotics #1
Baby101	101	pre eclampsia		Positive	No	Yes	pre eclampsia		Yes	penicillin
Baby102	102			Positive	Yes	Yes	bradycardia		Yes	penicillin
Baby103	103			Positive	Yes	Yes	vacuum delivery		Yes	penicillin
Baby104	104			Negative	Yes	No			No	
Baby105	105	IVF (in vitro fertilization)		Negative	Not Documented	Not Documented			No	
Baby106	106			Positive	Yes	No			Yes	vancomycin

Baby107	107			Negative	Yes	No			Yes	cephalexin
Baby108	108			Negative	No	Yes	failure to progress	STAT C-Section	No	
Baby109	109			Positive	Yes	No			Yes	penicillin
Baby110	110			Negative	Yes	No			No	
Baby111	111			Negative	Yes	No			No	
Baby112	112			Positive	Not Documented	No			No	
Baby113	113			Positive	Yes	No			Yes	penicillin
Baby114	114			Negative	Yes	No			No	
Baby115	115			Negative	Yes	No			No	
Baby116	116			Positive	No	No			No	
Baby117	117			Positive	Yes	No			Yes	penicillin
Baby118	118			Positive	No	No			Yes	penicillin
Baby119	119			Negative	Yes	No			No	
Baby120	120			Negative	No	No			No	
Baby121	121			Negative	Yes	No			No	
Baby122	122			Negative	Yes	No			No	
Baby123	123			Negative	Yes	No			Yes	gentamicin
Baby124	124	gestational diabetes		Negative	Yes	No			No	
Baby125	125			Negative	No	Yes	failure to progress		No	
Baby126	126			Negative	No	No			No	
Baby127	127	syncopal episodes		Negative	No	No			Yes	valacyclovir (antiviral)
Baby128	128			Negative	No	No			Yes	valacyclovir (antiviral)
Baby129	129			Negative	Yes	No			No	
Baby130	130	placenta previa		Negative	Yes	No			No	
Baby131	131			Negative	Yes	No			No	
Baby132	132			Negative	No	No			No	
Baby133	133			Negative	No	No			No	
Baby134	134			Negative	No	No			No	
Baby135	135			Positive	Yes	No			Yes	Unknown
Baby136	136	hypertension		Negative	Yes	No			No	
Baby201	201	SGA (small for gestational age)	placenta previa	Positive	No	No			Yes	penicillin
Baby202	202			Negative	Not Documented	No			No	
Baby203	203	gestational diabetes		Negative	No	No			No	
Baby204	204			Negative	No	No			No	
Baby205	205	gestational diabetes		Negative	Yes	No			No	
Baby206	206			Positive	Yes	No			Yes	Unknown
Baby207	207			Negative	Yes	No			No	
Baby208	208	SGA (small for gestational age)		Negative	Yes	No			No	
Baby209	209			Positive	Yes	No			Yes	penicillin
Baby210	210	breech baby		Positive	Yes	Yes	breech		No	
Baby211	211			Negative	Yes	No			No	
Baby212	212			Positive	Not Documented	No			Yes	penicillin
Baby213	213			Negative	Yes	Yes	failure to progress		No	
Baby214	214			Negative	No	No			Yes	Unknown
Baby215	215			Negative	Yes	No			No	
Baby216	216			Negative	Yes	No			No	
Baby217	217			Negative	Yes	No			Yes	oseltamivir (antiviral)
Baby218	218			Negative	No	No			No	
Baby219	219	placenta shifting		Negative	Yes	No			No	
Baby220	220	short cervix		Negative	No	No			No	
Baby221	221			Negative	Yes	No			No	
Baby222	222	pre eclampsia		Negative	No	No			No	
Baby223	223			Negative	Yes	No			No	
Baby224	224			Positive	Unknown	No			Yes	ampicillin
Baby225	225	LGA (large for gestational age)		Positive	No	No			Yes	
Baby226	226	hypertension		Negative	No	No			Yes	amoxicillin
Baby227	227			Negative	Yes	No			No	
Baby228	228			Negative	Yes	No			Yes	amoxicillin
Baby229	229			Positive	No	No			Yes	penicillin
Baby230	230			Positive	No	No			Yes	penicillin
Baby231	231	placenta previa		Negative	Yes	No			No	
Baby232	232			Unknown	Not Documented	No			No	
Baby233	233			Negative	Yes	No			No	
Baby234	234			Negative	Yes	Yes	shoulder dystocia		No	
Baby235	235			Positive	Yes	No			Yes	penicillin
Baby236	236			Negative	Yes	No			No	
Baby237	237			Positive	Yes	No			Yes	vancomycin
Baby238	238	IVF (in vitro fertilization)	hypothyrodism, took levothyroxine	Negative	Yes	No			No	
Baby239	239	gestational diabetes		Negative	No	No			No	
Baby240	240			Positive	No	No			Yes	penicillin
Baby241	241			Negative	No	No			Yes	Unknown
Baby242	242			Negative	No	No			No	
Baby243	243	gallstones		Negative	Yes	No			No	
Baby244	244			Positive	No	No			Yes	penicillin
Baby245	245			Negative	No	No			Yes	amoxicillin
Baby246	246	gestational diabetes		Positive	Yes	Yes	chorioamnionitis		No	
Baby247	247			Negative	Yes	No			No	
Baby248	248			Positive	No	No			Yes	penicillin
Baby249	249			Negative	No	Yes	breech		No	
Baby250	250	losing fluid		Unknown	No	No			Yes	Unknown
Baby251	251	gestational diabetes	hypertension	Positive	No	No			Yes	
Baby252	252	gestational diabetes		Positive	No	Yes	large baby	gestational diabetes	Yes	Unknown
Baby253	253	gestational diabetes		Negative	No	No			No	
Baby254	254			Negative	No	No			No	
Baby255	255	chronic hypertension		Negative	No	No			No	
Baby256	256			Positive	Yes	No			Yes	Unknown
Baby257	257			Positive	No	No			Yes	clindamycin
Baby258	258			Positive	No	No			Yes	penicillin
Baby259	259			Positive	Yes	No			Yes	penicillin
Baby260	260			Negative	No	No			No	
Baby261	261			Negative	Yes	No			No	
Baby262	262			Negative	Yes	No			No	
Baby263	263			Positive	No	No			Yes	penicillin
Baby264	264			Negative	Yes	Yes	Not Documented		No	
Baby265	265			Positive	No	No			Yes	Unknown

PrimaryKey	Subject ID	When	Reason	Mother Antibiotics #2	When.1	Reason.1	Mother Antibiotic #3	When.2	Reason.2	Has the mother received immunosupprasants?
Baby101	101	2/16/2018 0:00								No
Baby102	102	2/26/2018 0:00	GBS	cephalexin	08/26/17, 11/26/17	UTI	nitrofurantoin	5/26/2017	UTI	No
Baby103	103	2/26/2018 0:00	GBS							Yes
Baby104	104									No
Baby105	105									No
Baby106	106	4/6/2018 0:00	GBS							No
Baby107	107	Not Documented	Not Documented							No
Baby108	108									No
Baby109	109	4/20/2018 0:00	GBS							No
Baby110	110									No

Baby111	111									No
Baby112	112									No
Baby113	113	5/18/2018 0:00	GBS							No
Baby114	114									No
Baby115	115									No
Baby116	116									No
Baby117	117	6/15/2018 0:00	GBS							No
Baby118	118	6/28/2018 0:00	GBS							No
Baby119	119									No
Baby120	120									No
Baby121	121									No
Baby122	122									No
Baby123	123	7/12/2018 0:00	C-section	clindamycin	7/12/2018 0:00	C-Section				No
Baby124	124									No
Baby125	125									Yes
Baby126	126									No
Baby127	127	on and off	herpes							No
Baby128	128	12/5/2017 0:00	shingles							No
Baby129	129									No
Baby130	130									No
Baby131	131									No
Baby132	132									No
Baby133	133									No
Baby134	134									No
Baby135	135	12/6/2018 0:00	GBS							No
Baby136	136									No
Baby201	201	3/5/2018 0:00	GBS							No
Baby202	202									No
Baby203	203									No
Baby204	204									No
Baby205	205									No
Baby206	206	Not Documented	GBS							No
Baby207	207									No
Baby208	208									No
Baby209	209		GBS							No
Baby210	210									No
Baby211	211									No
Baby212	212		GBS							No
Baby213	213									No
Baby214	214	11/24/2017 0:00	UTI, sinusitis	Unknown	4/23/2018 0:00	UTI, sinusitis				No
Baby215	215									No
Baby216	216									No
Baby217	217	1/12/2018 0:00	Flu like symptoms							No
Baby218	218									No
Baby219	219									No
Baby220	220									No
Baby221	221									No
Baby222	222									No
Baby223	223									No
Baby224	224	5/3/2018 0:00	GBS							No
Baby225	225									No
Baby226	226	10/1/2018 0:00	strep throat							No
Baby227	227									No
Baby228	228	4/1/2018 0:00	sinusitis							No
Baby229	229	5/11/2018 0:00	GBS							No
Baby230	230	5/14/2018 0:00	GBS							No
Baby231	231									No
Baby232	232									No
Baby233	233									No
Baby234	234									No
Baby235	235	6/19/2018 0:00	GBS							No
Baby236	236									No
Baby237	237	6/22/2018 0:00	GBS							No
Baby238	238									No
Baby239	239									No
Baby240	240	7/3/2018 0:00	GBS							No
Baby241	241	3/1/2018 0:00	Tooth Infection							No
Baby242	242									No
Baby243	243									No
Baby244	244	7/19/2018 0:00	GBS							No
Baby245	245	7/10/2018 0:00	sinusitis							No
Baby246	246									No
Baby247	247									No
Baby248	248	8/30/2018 0:00	GBS							No
Baby249	249									No
Baby250	250	9/10/2018 0:00	C-Section							No
Baby251	251									No
Baby252	252	9/19/2018 0:00	C-Section							No
Baby253	253									No
Baby254	254									Yes
Baby255	255									No
Baby256	256	10/1/2018 0:00	GBS							No
Baby257	257	4/29/2018 0:00	Cyst	unknown	10/24/2018 0:00	GBS				No
Baby258	258	10/26/2018 0:00	GBS							No
Baby259	259	10/30/2018 0:00	GBS							Yes
Baby260	260									No
Baby261	261									No
Baby262	262									No
Baby263	263	Not Documented	GBS							No
Baby264	264									No
Baby265	265	11/15/2018 0:00	sinusitis							No

PrimaryKey	Subject ID	Immunosupprasant #1	When.3	Reason.3	How many siblings does the infant have?	Ages of siblings	Prone to Ear Infections (father)	Prone to Ear Infections (mother)	Prone to Ear Infections (siblings)	Prone to other Infections (father)
Baby101	101				1	11y				
Baby102	102				0					
Baby103	103	RhoGAM	2/27/2018	O type mismatch	0					
Baby104	104				1	2y		mother	siblings	
Baby105	105				3	20y, 18y, 12y	father		siblings	
Baby106	106				0					
Baby107	107				0					
Baby108	108				0					
Baby109	109				0					
Baby110	110				0					
Baby111	111				1	1y	father			
Baby112	112				3	7y, 3y, 1y	father			
Baby113	113				0		father	mother		
Baby114	114				1	2y				

Baby115	115				2	10y, 6y				
Baby116	116				4	20m, 18m, 11m, 17m				
Baby117	117				0			mother		
Baby118	118				1	5y, 2y	father	mother	siblings	
Baby119	119				1	2y				father
Baby120	120				0					
Baby121	121				1			mother	siblings	
Baby122	122				0		father			
Baby123	123				Not Documented					
Baby124	124				2	7y, 4y		mother		
Baby125	125	prednisone	8/1/2018	Gout	0					father
Baby126	126				1	8y			siblings	
Baby127	127				2	10y, 5y			siblings	
Baby128	128				2	14y, 5y			siblings	
Baby129	129				1	3y				
Baby130	130				1	2y	father		siblings	
Baby131	131				1	6y			siblings	
Baby132	132				0					
Baby133	133				2	Not Documented		mother		
Baby134	134				0		father			father
Baby135	135				1	4y				
Baby136	136				0					
Baby201	201				2	2y, 5y	father			
Baby202	202				1	1.5y		mother		
Baby203	203				1	1.5y				
Baby204	204				2					
Baby205	205				2	4.5y, 2y				
Baby206	206				4	12y, 10y, 8y, 7y			siblings	
Baby207	207				4	6y, 3y, 2y, 1y		mother		
Baby208	208				1	18y				
Baby209	209				0					
Baby210	210				2	4y twins	father			
Baby211	211				0			mother		
Baby212	212				1	2.5y	father	mother	siblings	
Baby213	213				2	9y, 6y				father
Baby214	214				1	3y		mother	siblings	
Baby215	215				0		father	mother		
Baby216	216				0			mother		
Baby217	217				0					
Baby218	218				5	7y, 13y, 14y, 2 others unsure			siblings	
Baby219	219				1	12y				
Baby220	220				1	9y	father			
Baby221	221				0		father			
Baby222	222				1	3y				
Baby223	223				2	7y, 5y		mother	siblings	
Baby224	224				1	3y				
Baby225	225				5	10y, 7y, 8y, 5y, 4y	father			
Baby226	226				2	10y - 9 months apart			siblings	
Baby227	227				2	7y, 5y			siblings	
Baby228	228				1	23m	father		siblings	father
Baby229	229				3	12y, 11y, 4y			siblings	
Baby230	230				0		father	mother		
Baby231	231				0					
Baby232	232				2	5y, 2y			siblings	
Baby233	233				1	6y			siblings	
Baby234	234				2	4y, 2y		mother	siblings	
Baby235	235				2	20m twins				
Baby236	236				2	7y, 4y	father			
Baby237	237				1	4.5y, 2y			siblings	
Baby238	238				2	16y, 18y			siblings	father
Baby239	239				2	18y			siblings	
Baby240	240				1	8y			siblings	
Baby241	241				1	deceased				
Baby242	242				0					
Baby243	243				1	2y		mother	siblings	
Baby244	244				0					
Baby245	245				2	12y, 2y		mother	siblings	father
Baby246	246				5	11y, 10y, 9y, 6y, 4y	father	mother		
Baby247	247				0					
Baby248	248				4	12y, 10y, 9y, 7y				
Baby249	249				2	13y, 12y		mother	siblings	
Baby250	250				2	3y, 9m			siblings	
Baby251	251				0					
Baby252	252				2	9y, 7y				
Baby253	253				1	2y	father			
Baby254	254	Unknown Steroid	9/14/2018	increase baby's lung development	0					
Baby255	255				0					
Baby256	256				0			mother		
Baby257	257				0					
Baby258	258				1	4y				
Baby259	259	Unknown	10/9/2018	pre-term labor	2	7y, 1y				
Baby260	260				3	9y, 7y, 1y			siblings	
Baby261	261				0					
Baby262	262				1	5y				
Baby263	263				0					
Baby264	264				1	1y		mother		
Baby265	265				0			mother		

PrimaryKey	Subject ID	Prone to other Infections (mother)	Prone to other Infections (siblings)	Prone to Autoimmune Disease (father)	Prone to Autoimmune Disease (mother)	Prone to Autoimmune Disease (siblings)	Prone to Atopy (father)	Prone to Atopy (mother)	Prone to Atopy (siblings)	Prone to Cancer (father)
Baby101	101						father			
Baby102	102									
Baby103	103									
Baby104	104								siblings	
Baby105	105					sibling			siblings	
Baby106	106						father			
Baby107	107									
Baby108	108						father	mother		
Baby109	109									
Baby110	110									
Baby111	111									
Baby112	112				mother		father	mother		
Baby113	113									
Baby114	114									
Baby115	115									
Baby116	116							mother		
Baby117	117									
Baby118	118						father			

Baby119	119							mother	siblings	
Baby120	120						father	mother		
Baby121	121									
Baby122	122									
Baby123	123									
Baby124	124									
Baby125	125	mother					father	mother		
Baby126	126						father		siblings	
Baby127	127			father				mother	siblings	
Baby128	128								siblings	
Baby129	129									
Baby130	130								siblings	
Baby131	131									
Baby132	132									
Baby133	133									
Baby134	134									
Baby135	135						father	mother	siblings	
Baby136	136						father	mother		
Baby201	201	mother						mother	siblings	
Baby202	202	mother								
Baby203	203									
Baby204	204									
Baby205	205								siblings	
Baby206	206			father	mother		father			
Baby207	207									
Baby208	208									
Baby209	209									
Baby210	210									
Baby211	211									
Baby212	212									
Baby213	213	mother						mother		
Baby214	214	mother							siblings	
Baby215	215						father	mother		
Baby216	216	mother						mother		
Baby217	217	mother					father	mother		
Baby218	218	mother								
Baby219	219									
Baby220	220	mother					father	mother		
Baby221	221									
Baby222	222									
Baby223	223									
Baby224	224							mother	siblings	
Baby225	225									
Baby226	226	mother							siblings	
Baby227	227		siblings						siblings	
Baby228	228							mother	siblings	
Baby229	229									
Baby230	230						father	mother		
Baby231	231	mother					father			
Baby232	232									
Baby233	233	mother								
Baby234	234									
Baby235	235									
Baby236	236									
Baby237	237									
Baby238	238									
Baby239	239								siblings	
Baby240	240							mother		
Baby241	241							mother		
Baby242	242									
Baby243	243						father		siblings	
Baby244	244									
Baby245	245								siblings	
Baby246	246							mother		
Baby247	247							mother		
Baby248	248		siblings						siblings	
Baby249	249						father			
Baby250	250							mother	siblings	
Baby251	251				mother			mother		
Baby252	252							mother	siblings	father
Baby253	253							mother		
Baby254	254						father			
Baby255	255									
Baby256	256	mother						mother		
Baby257	257						father	mother		
Baby258	258									
Baby259	259				mother				siblings	
Baby260	260									
Baby261	261									
Baby262	262									
Baby263	263									
Baby264	264									
Baby265	265									

PrimaryKey	Subject ID	Prone to Cancer (mother)	Prone to Cancer (siblings)	Prone to Other Condition?	Which other condition?	Are there pets at home?	Dog	Cat	Rodent	Fish
Baby101	101					Yes	1	1		
Baby102	102					No				
Baby103	103			father	G6PD deficiency	Yes	1			
Baby104	104					Yes	1			
Baby105	105					Yes	1			
Baby106	106					Yes	1	1		
Baby107	107					Yes	1			
Baby108	108					Yes		1		1
Baby109	109					Yes		1		
Baby110	110					Yes	1	1		
Baby111	111					Yes	1	1		
Baby112	112			mother	possible celiac -> being tested	No				
Baby113	113					Yes	1	1		
Baby114	114					Yes		1		
Baby115	115					No				
Baby116	116					Yes	1			
Baby117	117					Yes	1			
Baby118	118			mother	guillian-barre	Yes	1			1
Baby119	119					Yes	1	1		
Baby120	120					Yes		1		
Baby121	121					Yes	1			
Baby122	122					No				

Baby123	123					Yes	1	1		
Baby124	124					No				
Baby125	125			mother	migraines	Yes	1	1		
Baby126	126					Yes			1	
Baby127	127					Yes	1			
Baby128	128					No				
Baby129	129					Yes	1	1		
Baby130	130					Yes		1		
Baby131	131					Yes	1			
Baby132	132					No				
Baby133	133					Yes		1		
Baby134	134					Yes		1		1
Baby135	135					No				
Baby136	136					Yes	1	1		
Baby201	201					Yes	1			
Baby202	202					No				
Baby203	203					Yes	1			
Baby204	204					No				
Baby205	205					Yes	1			
Baby206	206					Yes	1			
Baby207	207					Yes	1			
Baby208	208					Yes	1	1		
Baby209	209					No				
Baby210	210					No				
Baby211	211					Yes		1		
Baby212	212					No				
Baby213	213					Yes		1		
Baby214	214					Yes		1		
Baby215	215					Yes	1	1		
Baby216	216					No				
Baby217	217					Yes	1	1		
Baby218	218					No				
Baby219	219					Yes		1		
Baby220	220					Yes	1			
Baby221	221					Yes	1			
Baby222	222					No				
Baby223	223					Yes	1	1		
Baby224	224					No				
Baby225	225					Yes			1	
Baby226	226					Yes	1	1		
Baby227	227			father	Migraine HA's and IBS + seizure disorder	No				
Baby228	228					Yes	1	1		
Baby229	229		siblings			Yes	1	1		
Baby230	230					Yes	1			
Baby231	231					Yes		1		
Baby232	232			father	COPD	No				
Baby233	233					Yes		1		
Baby234	234					Yes	1			
Baby235	235					No				
Baby236	236					No				
Baby237	237					Yes	1			
Baby238	238					No				
Baby239	239					Yes	1			
Baby240	240					No				
Baby241	241					Yes	1	1		
Baby242	242					Yes	1			
Baby243	243					No				
Baby244	244					Yes	1			
Baby245	245					Yes	1			
Baby246	246					Yes				
Baby247	247					Yes	1			
Baby248	248					Yes			1	
Baby249	249					Yes	1	1		
Baby250	250					No				
Baby251	251	mother				Yes		1		
Baby252	252			siblings	autism	Yes		1		
Baby253	253					Yes	1			
Baby254	254					Yes	1			
Baby255	255					No				
Baby256	256					No				
Baby257	257			father	bell's palsy	Yes		1		1
Baby258	258					No				
Baby259	259	mother				Yes		1		
Baby260	260					Yes	1	1		
Baby261	261					Yes	1	1		
Baby262	262					No				
Baby263	263					No				
Baby264	264					Yes		1		
Baby265	265					Yes		1		

PrimaryKey	Subject ID	Amphibian/Reptile	Bird	Other	Live on Farm?	Smokers at home?	Any medical conditions/signs/symptoms prior to study?	Condition #1	Past or Current?	Condition #2
Baby101	101				No	No	Yes	Nevus	Current	
Baby102	102				No	No	No			
Baby103	103				No	No	Yes	broken collar bone	Current	
Baby104	104				No	No	No			
Baby105	105			pigs	No	No	Yes	Jaundice	Current	
Baby106	106				No	No	No			
Baby107	107				No	No	Yes	Weight Loss	Current	Jaundice
Baby108	108				No	No	Yes	Umbilical cord granuloma	Current	
Baby109	109				No	Yes, outside	No			
Baby110	110				No	No	No			
Baby111	111	1			No	No	No			
Baby112	112				No	No	No			
Baby113	113	1			No	No	No			
Baby114	114				Yes	No	No			
Baby115	115				No	No	No			
Baby116	116				No	No	No			
Baby117	117				No	No	No			
Baby118	118				No	No	No			
Baby119	119				No	No	No			
Baby120	120				No	No	No			
Baby121	121				No	No	No			
Baby122	122				No	No	No			
Baby123	123				No	No	No			
Baby124	124				No	No	Yes	Jaundice	Current	
Baby125	125				No	Yes	No			
Baby126	126				No	No	No			

Baby127	127				No	No	No			
Baby128	128				No	No	No			
Baby129	129				No	No	No			
Baby130	130				No	No	No			
Baby131	131				No	No	No			
Baby132	132				No	No	No			
Baby133	133				No	No	No			
Baby134	134				No	No	Yes	milk protein allergy, bloody stool	Past	
Baby135	135				No	No	No			
Baby136	136				No	No	No			
Baby201	201				No	Yes	No			
Baby202	202				No	Yes, outside	Yes	Nasal Congestion	Current	
Baby203	203				No	No	No			
Baby204	204				No	No	No			
Baby205	205				No	No	No			
Baby206	206				No	No	No			
Baby207	207				No	No	No			
Baby208	208				No	Yes, outside	No			
Baby209	209				No	No	No			
Baby210	210				No	No	Yes	During ultrasound - Hip dysplasia	Past	
Baby211	211				No	No	No			
Baby212	212				No	No	No			
Baby213	213				No	Yes	No			
Baby214	214				No	Yes	No			
Baby215	215				No	No	No			
Baby216	216				No	No	No			
Baby217	217				No	No	No			
Baby218	218				No	No	No			
Baby219	219				No	Yes, outside	Yes	Candida skin infection	Current	
Baby220	220				No	Yes, outside	No			
Baby221	221				No	Yes	No			
Baby222	222				No	No	Yes	Erythema toxicum neonatorum	Current	
Baby223	223				No	Yes, outside	Yes	Erythema toxicum neonatorum		
Baby224	224				No	Yes	No			
Baby225	225				No	Yes	Yes	jaundice	Current	
Baby226	226				No	No	Yes	jaundice	Current	
Baby227	227				No	No	No			
Baby228	228				No	No	Yes	jaundice	Current	
Baby229	229				No	No	No			
Baby230	230				No	Yes, outside	No			
Baby231	231				No	Yes	No			
Baby232	232				No	No	No			
Baby233	233				No	Yes, outside	No			
Baby234	234				No	No	No			
Baby235	235				No	No	No			
Baby236	236				No	No	No			
Baby237	237		1		No	No	No			
Baby238	238				No	No	No			
Baby239	239				No	No	No			
Baby240	240				No	No	No			
Baby241	241				Yes	Yes, outside	No			
Baby242	242				No	No	Yes	Umbilical cord granuloma		
Baby243	243				No	No	No			
Baby244	244				No	No	No			
Baby245	245				No	No	Yes	hypoxia	Past	
Baby246	246	1			No	No	No			
Baby247	247				No	No	No			
Baby248	248				No	No	No			
Baby249	249				No	No	No			
Baby250	250				No	Yes	Yes	Polydactyly of left hand	Current	
Baby251	251				No	Yes	No			
Baby252	252				No	No	No			
Baby253	253				No	No	No			
Baby254	254				No	No	No			
Baby255	255				No	No	No			
Baby256	256				No	No	No			
Baby257	257				No	Yes	Yes	jaundice	Past	blocked tear duct
Baby258	258				No	No	No			
Baby259	259				No	No	No			
Baby260	260				No	No	No			
Baby261	261				No	No	No			
Baby262	262				No	Yes	No			
Baby263	263				No	Yes	Yes	jaundice	Past	
Baby264	264				No	Yes, Outside	No			
Baby265	265				No	No	No			

PrimaryKey	Subject ID	Past or Current?.1
Baby101	101	
Baby102	102	
Baby103	103	
Baby104	104	
Baby105	105	
Baby106	106	
Baby107	107	Current
Baby108	108	
Baby109	109	
Baby110	110	
Baby111	111	
Baby112	112	
Baby113	113	
Baby114	114	
Baby115	115	
Baby116	116	
Baby117	117	
Baby118	118	
Baby119	119	
Baby120	120	
Baby121	121	
Baby122	122	
Baby123	123	
Baby124	124	
Baby125	125	
Baby126	126	
Baby127	127	
Baby128	128	
Baby129	129	
Baby130	130	

Baby131	131	
Baby132	132	
Baby133	133	
Baby134	134	
Baby135	135	
Baby136	136	
Baby201	201	
Baby202	202	
Baby203	203	
Baby204	204	
Baby205	205	
Baby206	206	
Baby207	207	
Baby208	208	
Baby209	209	
Baby210	210	
Baby211	211	
Baby212	212	
Baby213	213	
Baby214	214	
Baby215	215	
Baby216	216	
Baby217	217	
Baby218	218	
Baby219	219	
Baby220	220	
Baby221	221	
Baby222	222	
Baby223	223	
Baby224	224	
Baby225	225	
Baby226	226	
Baby227	227	
Baby228	228	
Baby229	229	
Baby230	230	
Baby231	231	
Baby232	232	
Baby233	233	
Baby234	234	
Baby235	235	
Baby236	236	
Baby237	237	
Baby238	238	
Baby239	239	
Baby240	240	
Baby241	241	
Baby242	242	
Baby243	243	
Baby244	244	
Baby245	245	
Baby246	246	
Baby247	247	
Baby248	248	
Baby249	249	
Baby250	250	
Baby251	251	
Baby252	252	
Baby253	253	
Baby254	254	
Baby255	255	
Baby256	256	
Baby257	257	Current
Baby258	258	
Baby259	259	
Baby260	260	
Baby261	261	
Baby262	262	
Baby263	263	
Baby264	264	
Baby265	265	

Table S2. Cohort antibiotic usage.

PrimaryKey	BabyN	AntibioticN	Name	Reason	Start_Date	End_Date	Duration_(days)	DateOfBirth	AgeAtStart	AgeAtEnd	Route
Baby134_Antibiotic1	Baby134	1	piperacillin/ tazobactam	R/O sepsis	11/26/2018	11/28/2018	2	11/22/2018	4	6	IV
Baby134_Antibiotic2	Baby134	2	ampicillin	R/O sepsis	11/26/2018	11/27/2018	1	11/22/2018	4	5	IV
Baby134_Antibiotic3	Baby134	3	gentamicin	R/O sepsis	11/26/2018	11/27/2018	1	11/22/2018	4	5	IV
Baby134_Antibiotic4	Baby134	4	vancomycin	R/O sepsis	11/26/2018	11/27/2018	1	11/22/2018	4	5	IV
Baby235_Antibiotic1	Baby235	1	unknown anitbiotic(s)	R/O sepsis, later confirmed neg.	6/19/2018	Not Documented	Unknown	6/13/2018	6		
Baby245_Antibiotic1	Baby245	1	ampicillin	respiratory issues, R/O sepsis at birth	7/23/2018	7/26/2018	3	7/22/2018	1	4	IV
Baby245_Antibiotic2	Baby245	2	gentamicin	respiratory issues, R/O sepsis at birth	7/23/2018	7/26/2018	3	7/22/2018	1	4	IV
Baby246_Antibiotic1	Baby246	1	ampicillin	maternal chorioamnionitis	7/28/2018	7/31/2018	3	7/28/2018	0	3	IV
Baby263_Antibiotic1	Baby263	1	penicillin	GBS	Not Documented	Not Documented	Unknown	11/24/2018			oral
Baby102_Antibiotic1	Baby102	1	erythromycin	conjunctivitis	3/7/2018	3/14/2018	7	2/20/2018	15	22	topical
Baby103_Antibiotic1	Baby103	1	amoxicillin	Pneumonia	1/7/2020	1/17/2020	10	2/21/2018	685	695	oral
Baby105_Antibiotic1	Baby105	1	erythromycin	conjunctivitis	4/11/2018	4/18/2018	7	3/30/2018	12	19	topical
Baby106_Antibiotic1	Baby106	1	erythromycin	conjunctivitis	7/11/2018	7/18/2018	7	3/27/2018	106	113	topical
Baby106_Antibiotic2	Baby106	2	amoxicillin	AOM	1/16/2019	1/26/2019	10	3/27/2018	295	305	oral
Baby107_Antibiotic1	Baby107	1	tobramycin	conjunctivitis	7/10/2018	7/25/2018	15	4/2/2018	99	114	topical
Baby107_Antibiotic2	Baby107	2	amoxicillin	OME	12/12/2018	12/22/2018	10	4/2/2018	254	264	oral
Baby107_Antibiotic3	Baby107	3	amoxicillin	bronchiolitis	1/4/2019	1/11/2019	7	4/2/2018	277	284	oral
Baby107_Antibiotic4	Baby107	4	cefdinir	bronchilitis, pneumonia	1/11/2019	1/14/2019	3	4/2/2018	284	287	oral
Baby107_Antibiotic5	Baby107	5	cefdinir	Started to treat pneumonia, continued on treatment for AOM	1/14/2019	1/18/2019	4	4/2/2018	287	291	oral
Baby107_Antibiotic6	Baby107	6	oseltamivir (antiviral)	Urgent Care dx of flu	3/1/2019	3/3/2019	2	4/2/2018	333	335	
Baby107_Antibiotic7	Baby107	7	cefdinir	AOM	4/13/2019	4/23/2019	10	4/2/2018	376	386	oral
Baby107_Antibiotic8	Baby107	8	ceftriaxone	AOM	4/29/2019	4/30/2019	1	4/2/2018	392	393	IM
Baby108_Antibiotic1	Baby108	1	erythromycin	conjunctivitis	6/12/2018	6/19/2018	7	3/28/2018	76	83	topical
Baby108_Antibiotic2	Baby108	2	amoxicillin	AOM	4/4/2019	4/14/2019	10	3/28/2018	372	382	oral
Baby108_Antibiotic3	Baby108	3	cefdinir	AOM	6/27/2019	7/7/2019	10	3/28/2018	456	466	oral
Baby108_Antibiotic4	Baby108	4	ceftriaxone	AOM	7/11/2019	7/13/2019	2	3/28/2018	470	472	IM
Baby108_Antibiotic5	Baby108	5	ceftriaxone	AOM	7/13/2019	7/13/2019	0	3/28/2018	472	472	IM
Baby108_Antibiotic6	Baby108	6	cefdinir	AOM, Viral URI, perioral cyanosis	7/18/2019	7/25/2019	7	3/28/2018	477	484	oral
Baby108_Antibiotic7	Baby108	7	cefdinir	AOM	10/21/2019	10/28/2019	7	3/28/2018	572	579	oral
Baby108_Antibiotic8	Baby108	8	cefdinir	AOM	11/5/2019	11/15/2019	10	3/28/2018	587	597	oral
Baby108_Antibiotic9	Baby108	9	cefdinir	OME	1/23/2020	2/2/2020	10	3/28/2018	666	676	oral
Baby108_Antibiotic10	Baby108	10	tobramycin	conjunctivitis	1/23/2020	1/28/2020	5	3/28/2018	666	671	topical
Baby108_Antibiotic11	Baby108	11	cefdinir	AOM	2/5/2020	2/15/2020	10	3/28/2018	679	689	oral
Baby108_Antibiotic12	Baby108	12	cefdinir	AOM	2/17/2020	2/27/2020	10	3/28/2018	691	701	oral
Baby109_Antibiotic1	Baby109	1	erythromycin	conjunctivitis	5/4/2018	5/17/2018	13	4/17/2018	17	30	topical
Baby109_Antibiotic2	Baby109	2	amoxicillin	URI	11/26/2018	12/6/2018	10	4/17/2018	223	233	oral
Baby109_Antibiotic3	Baby109	3	cefdinir	URI	5/3/2019	5/8/2019	5	4/17/2018	381	386	oral
Baby109_Antibiotic4	Baby109	4	ceftriaxone	given in emergency department	5/8/2019	5/8/2019	0	4/17/2018	386	386	IM
Baby110_Antibiotic1	Baby110	1	erythromycin	conjunctivitis	10/3/2018	10/10/2018	7	5/7/2018	149	156	topical
Baby112_Antibiotic1	Baby112	1	amoxicillin	AOM, skin infection	12/20/2018	12/30/2018	10	5/3/2018	231	241	oral
Baby112_Antibiotic2	Baby112	2	amoxicillin	AOM	4/29/2019	5/8/2019	9	5/3/2018	361	370	oral
Baby112_Antibiotic3	Baby112	3	ofloxacin	AOM	5/13/2019	5/18/2019	5	5/3/2018	375	380	topical
Baby112_Antibiotic4	Baby112	4	cefdinir	AOM	5/13/2019	5/20/2019	7	5/3/2018	375	382	oral
Baby112_Antibiotic5	Baby112	5	amoxicillin	AOM	8/26/2019	9/5/2019	10	5/3/2018	480	490	oral
Baby113_Antibiotic1	Baby113	1	amoxicillin	AOM	9/19/2018	9/29/2018	10	5/15/2018	127	137	oral
Baby113_Antibiotic2	Baby113	2	erythromycin	conjunctivitis	8/24/2018	8/31/2018	7	5/15/2018	101	108	topical
Baby113_Antibiotic3	Baby113	3	tobramycin	conjunctivitis then stanosis of the nasolacrimal duct. Taken as needed (not regular schedule)	10/9/2018	11/17/2018	39	5/15/2018	147	186	topical
Baby114_Antibiotic1	Baby114	1	erythromycin	conjunctivitis	6/5/2018	6/12/2018	7	5/21/2018	15	22	topical
Baby114_Antibiotic2	Baby114	2	tobramycin	conjunctivitis	4/9/2019	4/14/2019	5	5/21/2018	323	328	topical
Baby114_Antibiotic3	Baby114	3	amoxicillin	AOM	5/22/2019	6/1/2019	10	5/21/2018	366	376	oral
Baby119_Antibiotic1	Baby119	1	amoxicillin	AOM, bullous myringitis	12/10/2019	12/20/2019	10	6/30/2018	528	538	oral
Baby121_Antibiotic1	Baby121	1	cephalexin	skin infection	12/6/2018	12/13/2018	7	6/30/2018	159	166	oral
Baby123_Antibiotic1	Baby123	1	erythromycin	delayed gastric emptying/delayed motility	11/27/2018	3/12/2019	105	7/8/2018	142	247	
Baby127_Antibiotic1	Baby127	1	amoxicillin	AOM, rule out Pertussis	11/9/2018	11/19/2018	10	8/31/2018	70	80	oral
Baby127_Antibiotic2	Baby127	2	cefdinir	AOM	12/10/2018	12/20/2018	10	8/31/2018	101	111	oral
Baby127_Antibiotic3	Baby127	3	amoxicillin clavulanate	AOM	1/7/2019	1/17/2019	10	8/31/2018	129	139	oral
Baby127_Antibiotic4	Baby127	4	cefdinir	UTI	10/19/2019	10/26/2019	7	8/31/2018	414	421	oral
Baby127_Antibiotic5	Baby127	5	amoxicillin	AOM	12/26/2019	1/4/2020	9	8/31/2018	482	491	oral
Baby127_Antibiotic6	Baby127	6	cefdinir	UTI	8/26/2020	9/5/2020	10	8/31/2018	726	736	oral
Baby128_Antibiotic1	Baby128	1	amoxicillin clavulanate	AOM	1/21/2019	1/31/2019	10	8/26/2018	148	158	oral
Baby128_Antibiotic2	Baby128	2	cefdinir	AOM	4/2/2019	4/12/2019	10	8/26/2018	219	229	oral
Baby128_Antibiotic3	Baby128	3	ceftriaxone	AOM	4/15/2019	4/15/2019	0	8/26/2018	232	232	IM
Baby128_Antibiotic4	Baby128	4	cefdinir	AOM	4/2/2019	4/12/2019	10	8/26/2018	219	229	oral
Baby128_Antibiotic5	Baby128	5	ceftriaxone	AOM	4/16/2019	4/17/2019	1	8/26/2018	233	234	IM
Baby128_Antibiotic6	Baby128	6	ofloxacin	Ear Infection w/ tubes	5/27/2019	6/2/2019	6	8/26/2018	274	280	topical
Baby130_Antibiotic1	Baby130	1	amoxicillin	AOM	8/24/2019	9/3/2019	10	10/27/2018	301	311	oral
Baby131_Antibiotic1	Baby131	1	amoxicillin clavulanate	AOM	5/1/2019	5/11/2019	10	11/8/2018	174	184	oral
Baby131_Antibiotic2	Baby131	2	cefdinir	AOM	7/1/2019	7/16/2019	15	11/8/2018	235	250	oral
Baby131_Antibiotic3	Baby131	3	amoxicillin clavulanate	AOM	8/7/2019	8/20/2019	13	11/8/2018	272	285	oral
Baby131_Antibiotic4	Baby131	4	amoxicillin clavulanate	AOM, Conjunctivitis	10/5/2019	10/15/2019	10	11/8/2018	331	341	oral
Baby131_Antibiotic5	Baby131	5	cefdinir	AOM	10/16/2019	10/26/2019	10	11/8/2018	342	352	oral
Baby131_Antibiotic6	Baby131	6	ciprofloxacin ear drops	AOM	1/27/2020	2/3/2020	7	11/8/2018	445	452	topical
Baby131_Antibiotic7	Baby131	7	ofloxacin ear drops	AOM	1/20/2020	1/27/2020	7	11/8/2018	438	445	topical
Baby133_Antibiotic1	Baby133	1	amoxicillin	AOM	2/1/2020	2/11/2020	10	11/19/2018	439	449	oral
Baby201_Antibiotic1	Baby201	1	amoxicillin	AOM	11/30/2018	12/10/2018	10	2/22/2018	281	291	oral
Baby202_Antibiotic1	Baby202	1	amoxicillin	AOM	7/26/2018	8/2/2018	7	2/27/2018	149	156	oral
Baby202_Antibiotic2	Baby202	2	amoxicillin	Pneumonia	1/23/2019	2/2/2019	10	2/27/2018	330	340	oral
Baby202_Antibiotic3	Baby202	3	amoxicillin clavulanate	AOM	4/18/2019	4/19/2019	1	2/27/2018	415	416	oral
Baby202_Antibiotic4	Baby202	4	cefdinir	AOM	4/19/2019	4/24/2019	5	2/27/2018	416	421	oral
Baby203_Antibiotic1	Baby203	1	amoxicillin	AOM	12/31/2018	1/10/2019	10	2/28/2018	306	316	oral
Baby204_Antibiotic1	Baby204	1	amoxicillin clavulanate	AOM	1/10/2019	1/20/2019	10	2/26/2018	318	328	oral
Baby204_Antibiotic2	Baby204	2	amoxicillin	AOM	12/19/2018	1/10/2019	22	2/26/2018	296	318	oral
Baby204_Antibiotic3	Baby204	3	amoxicillin	AOM	5/20/2019	5/23/2019	3	2/26/2018	448	451	oral
Baby204_Antibiotic4	Baby204	4	cefdinir	AOM	5/23/2019	6/2/2019	10	2/26/2018	451	461	oral
Baby204_Antibiotic5	Baby204	5	amoxicillin clavulanate	AOM	5/28/2019	6/7/2019	10	2/26/2018	456	466	oral
Baby204_Antibiotic6	Baby204	6	amoxicillin	AOM, acute pharyngitis	11/18/2019	11/28/2019	10	2/26/2018	630	640	oral
Baby204_Antibiotic7	Baby204	7	amoxicillin clavulanate	AOM	11/29/2019	12/9/2019	10	2/26/2018	641	651	oral
Baby205_Antibiotic1	Baby205	1	amoxicillin clavulanate	AOM	1/21/2019	1/31/2019	10	2/28/2018	327	337	oral
Baby205_Antibiotic2	Baby205	2	amoxicillin clavulanate	AOM	3/6/2019	3/16/2019	10	2/28/2018	371	381	oral
Baby205_Antibiotic3	Baby205	3	amoxicillin	AOM	10/28/2019	11/7/2019	10	2/28/2018	607	617	oral
Baby207_Antibiotic1	Baby207	1	amoxicillin	AOM	1/15/2019	1/25/2019	10	3/11/2018	310	320	oral
Baby207_Antibiotic2	Baby207	2	amoxicillin	OME, tonsillitis	11/30/2019	12/10/2019	10	3/11/2018	629	639	oral
Baby210_Antibiotic1	Baby210	1	cefdinir	misdiagnosed AOM at ER/Urgent Care	5/25/2019	5/26/2019	1	3/10/2018	441	442	oral
Baby211_Antibiotic1	Baby211	1	amoxicillin	AOM	12/8/2018	12/18/2018	10	3/11/2018	272	282	oral
Baby212_Antibiotic1	Baby212	1	amoxicillin clavulanate	AOM	12/18/2018	12/25/2018	7	3/18/2018	275	282	oral
Baby212_Antibiotic2	Baby212	2	cefdinir	AOM	1/15/2019	1/25/2019	10	3/18/2018	303	313	oral
Baby212_Antibiotic3	Baby212	3	amoxicillin clavulanate	AOM	1/28/2019	2/4/2019	7	3/18/2018	316	323	oral
Baby214_Antibiotic1	Baby214	1	amoxicillin	AOM	9/27/2018	10/7/2018	10	4/4/2018	176	186	oral
Baby214_Antibiotic2	Baby214	2	cefdinir	AOM	10/17/2018	10/23/2018	6	4/4/2018	196	202	oral
Baby214_Antibiotic3	Baby214	3	cefdinir	OME	11/15/2018	11/25/2018	10	4/4/2018	225	235	oral
Baby214_Antibiotic4	Baby214	4	amoxicillin clavulanate	via phone for ear pain, no diagnosis	10/26/2018	11/5/2018	10	4/4/2018	205	215	oral
Baby214_Antibiotic5	Baby214	5	cefdinir	OME	11/29/2018	12/8/2018	9	4/4/2018	239	248	oral
Baby214_Antibiotic6	Baby214	6	oseltamivir (antiviral)	URI	3/12/2019	3/17/2019	5	4/4/2018	342	347	
Baby214_Antibiotic7	Baby214	7	ciprofloxacin/ dexamethasone	externa OM	4/23/2019	4/28/2019	5	4/4/2018	384	389	oral
Baby214_Antibiotic8	Baby214	8	ciprofloxacin/ dexamethasone	otorrhea	5/21/2019	6/10/2019	20	4/4/2018	412	432	oral
Baby214_Antibiotic9	Baby214	9	tobramycin	conjunctivitis	6/12/2019	6/22/2019	10	4/4/2018	434	444	topical
Baby214_Antibiotic10	Baby214	10	ciprofloxacin/ dexamethasone	Not Documented	1/2/2020	1/9/2020	7	4/4/2018	638	645	oral
Baby214_Antibiotic11	Baby214	11	tobramycin	conjunctivitis	12/11/2019	12/18/2019	7	4/4/2018	616	623	topical
Baby214_Antibiotic12	Baby214	12	Tamiflu	prescribed w/o office visit	5/12/2020	5/17/2020	5	4/4/2018	769	774	
Baby215_Antibiotic1	Baby215	1	unkown	surgery-pyelplasty	2/6/2020	2/6/2020	0	3/29/2018	679	679	
Baby217_Antibiotic1	Baby217	1	cefdinir	UTI	10/14/2018	11/2/2018	19	4/8/2018	189	208	oral
Baby217_Antibiotic2	Baby217	2	mupirocin	skin infection	4/2/2019	4/9/2019	7	4/8/2018	359	366	topical
Baby218_Antibiotic1	Baby218	1	amoxicillin	AOM	9/13/2018	9/23/2018	10	4/3/2018	163	173	oral
Baby219_Antibiotic1	Baby219	1	amoxicillin	AOM	12/27/2018	1/6/2019	10	4/10/2018	261	271	oral
Baby219_Antibiotic2	Baby219	2	polymyxin B sulfate/ trimethoprim	conjunctivitis	12/5/2018	12/12/2018	7	4/10/2018	239	246	topical
Baby220_Antibiotic1	Baby220	1	polymyxin B sulfate/ trimethoprim	conjunctivitis	5/15/2018	5/21/2018	6	4/13/2018	32	38	topical
Baby220_Antibiotic2	Baby220	2	polymyxin B sulfate/ trimethoprim	conjunctivitis	7/12/2018	7/19/2018	7	4/13/2018	90	97	topical
Baby220_Antibiotic3	Baby220	3	amoxicillin	AOM	12/10/2018	12/20/2018	10	4/13/2018	241	251	oral
Baby220_Antibiotic4	Baby220	4	amoxicillin clavulanate	AOM	12/27/2018	1/6/2019	10	4/13/2018	258	268	oral
Baby220_Antibiotic5	Baby220	5	oseltamivir (antiviral)	Influenza A	3/14/2019	3/19/2019	5	4/13/2018	335	340	
Baby223_Antibiotic1	Baby223	1	amoxicillin clavulanate	AOM, conjunctivitis	6/11/2019	6/21/2019	10	4/27/2018	410	420	oral
Baby224_Antibiotic1	Baby224	1	cefdinir	pyelonephritis	1/5/2019	1/7/2019	2	4/22/2018	258	260	oral
Baby226_Antibiotic1	Baby226	1	ciprofloxacin/ dexamethasone	otorrhea	10/15/2018	10/22/2018	7	5/3/2018	165	172	oral
Baby226_Antibiotic2	Baby226	2	erythromycin	conjunctivitis	5/15/2019	5/21/2019	6	5/3/2018	377	383	topical
Baby226_Antibiotic3	Baby226	3	amoxicillin clavulanate	AOM	5/24/2019	5/29/2019	5	5/3/2018	386	391	oral
Baby226_Antibiotic4	Baby226	4	polymyxin B sulfate/ trimethoprim	conjunctivitis	5/24/2019	5/29/2019	5	5/3/2018	386	391	topical
Baby226_Antibiotic5	Baby226	5	amoxicillin clavulanate	AOM	10/21/2019	10/26/2019	5	5/3/2018	536	541	oral

Baby227_Antibiotic1	Baby227	1	ceftriaxone	UTI	9/16/2018	9/16/2018	0	4/30/2018	139	139	IM
Baby227_Antibiotic2	Baby227	2	Bactrim (timethoprim/sulfamethaxazole)	UTI	9/16/2018	9/23/2018	7	4/30/2018	139	146	oral
Baby227_Antibiotic3	Baby227	3	amoxicillin	AOM	3/29/2019	4/8/2019	10	4/30/2018	333	343	oral
Baby227_Antibiotic4	Baby227	4	cefdinir	AOM	4/18/2019	4/28/2019	10	4/30/2018	353	363	oral
Baby228_Antibiotic1	Baby228	1	erythromycin	conjunctivitis	11/1/2018	11/11/2018	10	5/7/2018	178	188	topical
Baby228_Antibiotic2	Baby228	2	polymyxin B sulfate/ trimethoprim	conjunctivitis	11/1/2018	11/11/2018	10	5/7/2018	178	188	topical
Baby228_Antibiotic3	Baby228	3	amoxicillin	AOM	5/6/2019	5/16/2019	10	5/7/2018	364	374	oral
Baby228_Antibiotic4	Baby228	4	polymyxin B sulfate/ trimethoprim	conjunctivitis	10/18/2019	10/25/2019	7	5/7/2018	529	536	topical
Baby228_Antibiotic5	Baby228	5	amoxicillin clavulanate	AOM	1/24/2020	1/29/2020	5	5/7/2018	627	632	oral
Baby228_Antibiotic6	Baby228	6	cefdinir	AOM	2/4/2020	2/9/2020	5	5/7/2018	638	643	oral
Baby229_Antibiotic1	Baby229	1	amoxicillin clavulanate	AOM	2/7/2019	2/17/2019	10	4/29/2018	284	294	oral
Baby229_Antibiotic2	Baby229	2	amoxicillin clavulanate	AOM	3/6/2019	3/13/2019	7	4/29/2018	311	318	oral
Baby229_Antibiotic3	Baby229	3	polymyxin B sulfate/ trimethoprim	conjunctivitis	6/5/2019	6/12/2019	7	4/29/2018	402	409	topical
Baby230_Antibiotic1	Baby230	1	amoxicillin clavulanate	AOM	1/7/2019	1/17/2019	10	5/10/2018	242	252	oral
Baby230_Antibiotic2	Baby230	2	amoxicillin clavulanate	AOM	4/29/2019	5/9/2019	10	5/10/2018	354	364	oral
Baby230_Antibiotic3	Baby230	3	amoxicillin	AOM	4/23/2019	4/29/2019	6	5/10/2018	348	354	oral
Baby230_Antibiotic4	Baby230	4	amoxicillin clavulanate	AOM, conjunctivitis	7/10/2019	7/20/2019	10	5/10/2018	426	436	oral
Baby230_Antibiotic5	Baby230	5	cefdinir	AOM	7/22/2019	8/1/2019	10	5/10/2018	438	448	oral
Baby230_Antibiotic6	Baby230	6	amoxicillin	AOM	9/29/2019	9/30/2019	1	5/10/2018	507	508	oral
Baby230_Antibiotic7	Baby230	7	amoxicillin clavulanate	AOM	9/30/2019	10/10/2019	10	5/10/2018	508	518	oral
Baby231_Antibiotic1	Baby231	1	amoxicillin	AOM, Urgent Care	7/22/2019	8/1/2019	10	5/9/2018	439	449	oral
Baby231_Antibiotic2	Baby231	2	amoxicillin	AOM	2/12/2020	2/22/2020	10	5/9/2018	644	654	oral
Baby233_Antibiotic1	Baby233	1	amoxicillin	AOM	12/10/2018	12/20/2018	10	5/21/2018	203	213	oral
Baby233_Antibiotic2	Baby233	2	amoxicillin clavulanate	AOM	12/27/2018	1/5/2019	9	5/21/2018	220	229	oral
Baby233_Antibiotic3	Baby233	3	amoxicillin clavulanate	AOM, conjunctivitis	4/6/2019	4/13/2019	7	5/21/2018	320	327	oral
Baby233_Antibiotic4	Baby233	4	cefdinir	AOM, conjunctivitis	4/13/2019	4/23/2019	10	5/21/2018	327	337	oral
Baby233_Antibiotic5	Baby233	5	polymyxin B sulfate/ trimethoprim	conjunctivitis	4/13/2019	4/18/2019	5	5/21/2018	327	332	topical
Baby233_Antibiotic6	Baby233	6	bacitracin	conjunctivitis	4/6/2019	4/11/2019	5	5/21/2018	320	325	topical
Baby234_Antibiotic1	Baby234	1	cefdinir	AOM	2/5/2019	2/15/2019	10	6/15/2018	235	245	oral
Baby234_Antibiotic2	Baby234	2	cefdinir	OME	1/15/2019	2/5/2019	21	6/15/2018	214	235	oral
Baby234_Antibiotic3	Baby234	3	amoxicillin clavulanate	AOM	12/26/2018	1/5/2019	10	6/15/2018	194	204	oral
Baby234_Antibiotic4	Baby234	4	ofloxacin	Ear tubes placed	6/28/2019	7/5/2019	7	6/15/2018	378	385	topical
Baby234_Antibiotic5	Baby234	5	amoxicillin	Pneumonia	1/11/2020	1/18/2020	7	6/15/2018	575	582	oral
Baby235_Antibiotic2	Baby235	2	amoxicillin clavulanate	AOM	1/7/2019	1/12/2019	5	6/13/2018	208	213	oral
Baby235_Antibiotic3	Baby235	3	ciprofloxacin/ dexamethasone	AOM, ear drainage	12/9/2019	12/16/2019	7	6/13/2018	544	551	oral
Baby235_Antibiotic4	Baby235	4	cefdinir	otorrhea	12/12/2019	12/22/2019	10	6/13/2018	547	557	oral
Baby235_Antibiotic5	Baby235	5	ofloxacin	otorrhea	12/12/2019	12/17/2019	5	6/13/2018	547	552	topical
Baby235_Antibiotic6	Baby235	6	ciprofloxacin/ dexamethasone	ear drainage, PE Tubes	2/7/2020	2/14/2020	7	6/13/2018	604	611	oral
Baby235_Antibiotic7	Baby235	7	cefdinir	AOM	3/24/2020	4/3/2020	10	6/13/2018	650	660	oral
Baby235_Antibiotic8	Baby235	8	ofloxacin ear drops	pulling on ears (no office visit)	3/21/2020	3/22/2020	1	6/13/2018	647	648	topical
Baby235_Antibiotic9	Baby235	9	ofloxacin ear drops	unknown (no office visit)	5/18/2020	5/25/2020	7	6/13/2018	705	712	topical
Baby236_Antibiotic1	Baby236	1	polymyxin B sulfate/ trimethoprim	conjunctivitis	11/23/2018	11/28/2018	5	6/12/2018	164	169	topical
Baby236_Antibiotic2	Baby236	2	cefdinir	AOM	12/27/2018	1/1/2019	5	6/12/2018	198	203	oral
Baby236_Antibiotic3	Baby236	3	ciprofloxacin/ dexamethasone	AOM	4/14/2019	4/19/2019	5	6/12/2018	306	311	oral
Baby236_Antibiotic4	Baby236	4	amoxicillin	AOM	4/16/2019	4/26/2019	10	6/12/2018	308	318	oral
Baby236_Antibiotic5	Baby236	5	ciprofloxacin/ dexamethasone	AOM	4/14/2019	4/21/2019	7	6/12/2018	306	313	oral
Baby236_Antibiotic6	Baby236	6	amoxicillin clavulanate	AOM, conjunctivitis	5/15/2019	5/25/2019	10	6/12/2018	337	347	oral
Baby236_Antibiotic7	Baby236	7	amoxicillin clavulanate	AOM	5/27/2019	6/6/2019	10	6/12/2018	349	359	oral
Baby236_Antibiotic8	Baby236	8	tobramycin	conjunctivitis	5/27/2019	6/6/2019	10	6/12/2018	349	359	topical
Baby236_Antibiotic9	Baby236	9	cefdinir	AOM	6/14/2019	6/24/2019	10	6/12/2018	367	377	oral
Baby236_Antibiotic10	Baby236	10	polymyxin B sulfate/ trimethoprim	conjunctivitis	7/10/2019	7/15/2019	5	6/12/2018	393	398	topical
Baby238_Antibiotic1	Baby238	1	unknown in IV (at Strong Memorial Hospital)	Viral meningitis (to rule out bacterial infection)	7/13/2018	7/14/2018	1	6/20/2018	23	24	IV
Baby238_Antibiotic2	Baby238	2	polymyxin B sulfate/ trimethoprim	dacryostenosis left eye	9/19/2018	9/24/2018	5	6/20/2018	91	96	topical
Baby239_Antibiotic1	Baby239	1	erythromycin	conjunctivitis	1/2/2020	1/6/2020	4	6/24/2018	557	561	topical
Baby239_Antibiotic2	Baby239	2	polymyxin B sulfate/ trimethoprim	conjunctivitis	1/6/2020	1/13/2020	7	6/24/2018	561	568	topical
Baby239_Antibiotic3	Baby239	3	erythromycin	conjunctivitis	1/6/2020	1/11/2020	5	6/24/2018	561	566	topical
Baby240_Antibiotic1	Baby240	1	amoxicillin clavulanate	AOM	4/18/2019	4/28/2019	10	6/21/2018	301	311	oral
Baby240_Antibiotic2	Baby240	2	polymyxin B sulfate/ trimethoprim	conjunctivitis	4/18/2019	4/23/2019	5	6/21/2018	301	306	topical
Baby240_Antibiotic3	Baby240	3	amoxicillin clavulanate	AOM	7/19/2019	7/24/2019	5	6/21/2018	393	398	oral
Baby240_Antibiotic4	Baby240	4	amoxicillin clavulanate	AOM, OME	11/26/2019	12/6/2019	10	6/21/2018	523	533	oral
Baby241_Antibiotic1	Baby241	1	amoxicillin	AOM	1/2/2020	1/12/2020	10	6/24/2018	557	567	oral
Baby241_Antibiotic2	Baby241	2	amoxicillin clavulanate	AOM, conjunctivitis	7/9/2019	7/19/2019	10	6/24/2018	380	390	oral
Baby241_Antibiotic3	Baby241	3	amoxicillin	AOM	11/7/2019	11/27/2019	20	6/24/2018	501	521	oral
Baby241_Antibiotic4	Baby241	4	amoxicillin	AOM	5/3/2019	5/13/2019	10	6/24/2018	313	323	oral
Baby241_Antibiotic5	Baby241	5	cefdinir	AOM	1/13/2020	1/21/2020	8	6/24/2018	568	576	oral
Baby241_Antibiotic6	Baby241	6	ceftriaxone	AOM	1/21/2020	1/21/2020	0	6/24/2018	576	576	IM
Baby243_Antibiotic1	Baby243	1	amoxicillin	AOM	12/23/2018	12/27/2018	4	7/11/2018	165	169	oral
Baby243_Antibiotic2	Baby243	2	amoxicillin	AOM	10/16/2019	10/26/2019	10	7/11/2018	462	472	oral
Baby245_Antibiotic3	Baby245	3	amoxicillin	AOM	4/16/2019	4/26/2019	10	7/22/2018	268	278	oral
Baby245_Antibiotic4	Baby245	4	amoxicillin	AOM	6/25/2019	7/5/2019	10	7/22/2018	338	348	oral
Baby245_Antibiotic5	Baby245	5	amoxicillin clavulanate	AOM	7/19/2019	7/24/2019	5	7/22/2018	362	367	oral
Baby245_Antibiotic6	Baby245	6	amoxicillin	AOM	1/11/2020	1/21/2020	10	7/22/2018	538	548	oral
Baby245_Antibiotic7	Baby245	7	amoxicillin clavulanate	strep throat	2/17/2020	2/23/2020	6	7/22/2018	575	581	oral
Baby245_Antibiotic8	Baby245	8	Bactrim (timethoprim/sulfamethaxazole)	cellulitis and abscess	2/24/2020	3/5/2020	10	7/22/2018	582	592	
Baby247_Antibiotic1	Baby247	1	tamiflu	Influenza A	2/25/2020	3/1/2020	5	8/24/2018	550	555	
Baby248_Antibiotic1	Baby248	1	erythromycin	conjunctivitis	8/30/2018	9/25/2018	26	8/19/2018	11	37	topical
Baby249_Antibiotic1	Baby249	1	amoxicillin clavulanate	AOM	1/4/2019	1/14/2019	10	8/24/2018	133	143	oral
Baby249_Antibiotic2	Baby249	2	amoxicillin	AOM	1/30/2019	2/9/2019	10	8/24/2018	159	169	oral
Baby249_Antibiotic3	Baby249	3	Tamiflu	Influenza A	3/4/2019	3/9/2019	5	8/24/2018	192	197	
Baby249_Antibiotic4	Baby249	4	amoxicillin	AOM	3/31/2020	4/10/2020	10	8/24/2018	585	595	oral
Baby250_Antibiotic1	Baby250	1	cephalexin	Insect Bite	2/14/2019	2/24/2019	10	9/5/2018	162	172	oral
Baby250_Antibiotic2	Baby250	2	amoxicillin	AOM	7/28/2019	7/31/2019	3	9/5/2018	326	329	oral
Baby250_Antibiotic3	Baby250	3	cefdinir	AOM, switched due to allergic reaction to amoxicillin	7/31/2019	8/3/2019	3	9/5/2018	329	332	oral
Baby250_Antibiotic4	Baby250	4	azithromycin	AOM	9/23/2019	9/28/2019	5	9/5/2018	383	388	oral
Baby250_Antibiotic5	Baby250	5	azithromycin	AOM	10/19/2019	10/24/2019	5	9/5/2018	409	414	oral
Baby250_Antibiotic6	Baby250	6	azithromycin	AOM	1/31/2020	2/3/2020	3	9/5/2018	513	516	oral
Baby250_Antibiotic7	Baby250	7	ciprofloxacin/ dexamethasone	AOM	1/31/2020	2/5/2020	5	9/5/2018	513	518	oral
Baby250_Antibiotic8	Baby250	8	azithromycin	strep throat	3/25/2020	3/30/2020	5	9/5/2018	567	572	oral
Baby252_Antibiotic1	Baby252	1	Tamiflu	Influenza	12/8/2019	12/12/2019	4	9/6/2018	458	462	
Baby252_Antibiotic2	Baby252	2	polymyxin B sulfate/ trimethoprim	conjunctivitis	3/11/2020	3/21/2020	10	9/6/2018	552	562	topical
Baby253_Antibiotic1	Baby253	1	oseltamivir (antiviral)	URI	2/20/2019	2/25/2019	5	9/27/2018	146	151	
Baby254_Antibiotic1	Baby254	1	amoxicillin	AOM	4/2/2019	4/12/2019	10	9/28/2018	186	196	oral
Baby254_Antibiotic2	Baby254	2	amoxicillin clavulanate	pneumonia, AOM	11/22/2019	11/25/2019	3	9/28/2018	420	423	oral
Baby254_Antibiotic3	Baby254	3	amoxicillin	AOM	11/18/2019	11/22/2019	4	9/28/2018	416	420	oral
Baby254_Antibiotic4	Baby254	4	cefdinir	AOM	11/25/2019	12/11/2019	16	9/28/2018	423	439	oral
Baby254_Antibiotic5	Baby254	5	cefdinir	AOM	1/3/2020	1/13/2020	10	9/28/2018	462	472	oral
Baby254_Antibiotic6	Baby254	6	cefdinir	AOM	1/3/2020	1/13/2020	10	9/28/2018	462	472	oral
Baby256_Antibiotic1	Baby256	1	amoxicillin clavulanate	AOM	2/1/2019	2/11/2019	10	10/2/2018	122	132	oral
Baby256_Antibiotic2	Baby256	2	amoxicillin	AOM	5/17/2019	5/27/2019	10	10/2/2018	227	237	oral
Baby256_Antibiotic3	Baby256	3	amoxicillin	AOM	10/18/2019	10/28/2019	10	10/2/2018	381	391	oral
Baby256_Antibiotic4	Baby256	4	amoxicillin	RSV, AOM, Bronchiolitis	12/26/2019	1/5/2020	10	10/2/2018	450	460	oral
Baby256_Antibiotic5	Baby256	5	amoxicillin clavulanate	AOM	1/15/2020	1/25/2020	10	10/2/2018	470	480	oral
Baby256_Antibiotic6	Baby256	6	cefdinir	AOM	3/16/2020	3/18/2020	2	10/2/2018	531	533	oral
Baby256_Antibiotic7	Baby256	7	amoxicillin clavulanate	cellulitis	3/18/2020	3/28/2020	10	10/2/2018	533	543	oral
Baby256_Antibiotic8	Baby256	8	Unknown	AOM; Given at ER, not in the medical record	10/7/2020	10/17/2020	10	10/2/2018	736	746	
Baby258_Antibiotic1	Baby258	1	amoxicillin	AOM	3/28/2019	4/7/2019	10	10/23/2018	156	166	oral
Baby258_Antibiotic2	Baby258	2	amoxicillin	AOM	12/4/2019	12/14/2019	10	10/23/2018	407	417	oral
Baby259_Antibiotic1	Baby259	1	amoxicillin	AOM, URI	4/21/2019	5/1/2019	10	10/26/2018	177	187	oral
Baby259_Antibiotic2	Baby259	2	oseltamivir (antiviral)	AOM, URI	4/21/2019	5/1/2019	10	10/26/2018	177	187	
Baby260_Antibiotic1	Baby260	1	polymyxin B sulfate/ trimethoprim	conjunctivitis	11/20/2018	11/27/2018	7	11/5/2018	15	22	topical
Baby261_Antibiotic1	Baby261	1	amoxicillin	Bronchiolitis	4/8/2019	4/13/2019	5	11/1/2018	158	163	oral
Baby261_Antibiotic2	Baby261	2	amoxicillin clavulanate	pyoderma	10/21/2019	10/28/2019	7	11/1/2018	354	361	oral
Baby261_Antibiotic3	Baby261	3	Bactrim (timethoprim/sulfamethaxazole)	pyoderma	10/21/2019	10/28/2019	7	11/1/2018	354	361	topical
Baby261_Antibiotic4	Baby261	4	polymyxin B sulfate/ trimethoprim	conjunctivitis	11/6/2019	11/13/2019	7	11/1/2018	370	377	topical
Baby261_Antibiotic5	Baby261	5	amoxicillin	AOM	1/30/2020	2/9/2020	10	11/1/2018	455	465	oral
Baby261_Antibiotic6	Baby261	6	amoxicillin clavulanate	dog bite, had stitches	4/26/2020	5/4/2020	8	11/1/2018	542	550	oral
Baby265_Antibiotic1	Baby265	1	erythromycin	skin infection	12/28/2018	1/2/2019	5	12/6/2018	22	27	topical

Table S3. Cohort vaccine respnse status and antibiotics at birth.

BabyN	NVR/LVR	Antibiotics at Birth
Baby106	NVR	No
Baby107	NVR	No
Baby108	LVR	No
Baby109	NVR	No
Baby110	NVR	No
Baby113	NVR	No
Baby114	NVR	No
Baby115	NVR	No
Baby117	NVR	No
Baby118	LVR	No
Baby119	NVR	No
Baby121	NVR	No
Baby122	NVR	No
Baby123	NVR	No
Baby125	NVR	No
Baby127	LVR	No
Baby129	LVR	No
Baby130	NVR	No
Baby131	NVR	No
Baby133	NVR	No
Baby134	LVR	Yes
Baby136	LVR	No
Baby201	NVR	No
Baby202	NVR	No
Baby204	LVR	No
Baby205	NVR	No
Baby208	NVR	No
Baby209	NVR	No
Baby210	NVR	No
Baby211	NVR	No
Baby212	NVR	No
Baby214	NVR	No
Baby215	NVR	No
Baby217	NVR	No
Baby218	NVR	No
Baby219	NVR	No
Baby220	NVR	No
Baby221	NVR	No
Baby223	NVR	No
Baby224	NVR	No
Baby226	NVR	No
Baby227	NVR	No
Baby228	NVR	No
Baby229	NVR	No
Baby230	LVR	No
Baby231	NVR	No
Baby233	NVR	No
Baby234	LVR	No
Baby235	NVR	Yes
Baby237	NVR	No
Baby238	NVR	No
Baby239	NVR	No
Baby240	LVR	No
Baby243	NVR	No
Baby245	LVR	Yes
Baby246	LVR	Yes
Baby247	NVR	No
Baby249	NVR	No
Baby250	NVR	No
Baby251	NVR	No
Baby252	NVR	No
Baby253	NVR	No
Baby254	NVR	No
Baby255	NVR	No
Baby256	NVR	No
Baby258	NVR	No
Baby259	NVR	No
Baby260	NVR	No
Baby261	NVR	No
Baby263	NVR	Yes
Baby264	NVR	No
Baby265	NVR	No

Table S4. HPLC method for detection of central carbon metabolites.

LC Conditions					
Column	Agilent ZORBAX RRHD Extend-C18, 2.1 × 150 mm, 1.8 µm				
Guard column	ZORBAX Eclipse Plus C18, 2.1 mm, 1.8 µm, UHPLC guard column				
Column temperature	40 ^oC				
Mobile phase	(A) Water:Methanol (97:3) with 15 mM glacial acetic acid and 10 mM tributylamine<br>(B) Methanol with 15 mM glacial acetic acid and 10 mM tributylamine<br>(D) Acetonitrile				
Gradient program	Time (min)	% A	% B	%D	Flow (mL/min)
	0	100	0	0	0.25
	2.5	100	0	0	0.25
	7.5	80	20	0	0.25
	13.00	55	45	0	0.25
	20.00	1	99	0	0.25
	24.00	1	99	0	0.25
	24.05	1	0	99	0.25
	27.00	1	0	99	0.25
	27.50	1	0	99	0.8
	31.35	1	0	99	0.8
	31.50	1	0	99	0.6
	32.25	100	0	0	0.4
	39.90	100	0	0	0.4
	40.00	100	0	0	0.25
MS Ionization mode	ESI negative				

Table S5. HPLC method for detection of bile acids.

LC Conditions				
Column	Agilent Poroshell EC-C18, 2.1 × 150 mm, 2.7 μm			
Guard column	Agilent Poroshell EC-C18, 2.1 × 5 mm, 2.7 μm, UHPLC guard column			
Column temperature	45 ^{o}C			
Mobile phase	(A) 0.1% formic acid + 20 mM ammonium acetate in water<br>(B) 0.1% formic acid in acetone			
Gradient program	Time (min)	% A	% B	Flow (mL/min)
	0.00	68	32	0.2
	6.00	68	32	0.2
	6.01	68	32	0.4
	25.00	35	65	0.4
	25.10	2	98	0.4
	27.10	2	98	0.4
	27.11	68	32	0.4
MS Ionization mode	ESI positive			

Table S6. HPLC method for additional central carbon metabolites.

LC Conditions				
Column	Agilent Zorbax SB-C18, 2.1 x 50 mm, 1.8 µm			
Column temperature	30 ^{o}C			
Mobile phase	(A) 0.1% formic acid in water<br>(B) 0.1% formic acid in acetonitrile			
Gradient program	Time (min)	% A	% B	Flow (mL/min)
	0.00	90	10	0.4
	1.00	90	10	0.4
	2.50	75	25	0.4
	3.00	0	100	0.4
	4.50	0	100	0.4
	4.60	90	10	0.4
MS Ionization mode	ESI positive			

Table S7. Metabolites detected by LC/MS QQQ analysis.

Compound name	CAS ID	Compound name	CAS ID
2-3-Dihydroxyisovalerate	1756-18-9	L-Glutamine	56-85-9
2-Deoxycytidine 5-diphosphate	4682-43-3	L-Histidine	71-00-1
2-Deoxyuridine	951-78-0	L-Isoleucine	61-90-5
2-Methyl-1-butanol	1565-80-6	L-Kynurenine	2922-83-0
2-Phosphoglyceric acid	2553-59-5	L-Leucine	61-90-5
3-Indoleacetic acid	87-51-4	L-Methionine	63-68-3
3-phenyllactic acid	828-01-3	L-Phenylalanine	63-91-2
4-Hydroxybenzoic acid	99-96-7	L-Proline	147-85-3
4-Hydroxyphenylacetic acid	156-38-7	L-Serine	56-45-1
4-Methyl-2-oxovaleric acid	816-66-0	L-Sorbose	87-79-6
4-Pyridoxic acid	82-82-6	L-Threonine	72-19-5
5-Hydroxyindoleacetic acid	54-16-0	L-Tryptophan	73-22-3
7-ketodeoxycholic Acid	911-40-0	L-Tyrosine	60-18-4
Adenosine	58-61-7	Maleic acid	110-16-7
Allantoin	97-59-6	Melibiose	5340-95-4
alpha-D(+)Mannose 1-phosphate	27251-84-9	Mevalonic acid	150-97-0
alpha-Ketoglutaric acid	328-50-7	myo-Inositol	87-89-8
Cholic acid	81-25-4	N-Acetyl D-galactosamine	14215-68-0
Chenodeoxycholic acid	474-25-9	N-Acetyl-alpha-D-glucosamine 1-phosphate	28446-21-1
cis-Aconitic acid	585-84-2	N-Acetylneuraminic acid	131-48-6
Creatine	57-00-1	N-Carbamyl-L-glutamic acid	1188-38-1
Creatinine	60-27-5	Nicotinic acid	59-67-6
Deoxycholic acid	83-44-3	Orotic acid	65-86-1
Deoxycytidine 5-triphosphate	2056-98-6	Oxamic acid	471-47-6
DL-2-Aminoadipic acid	542-32-5	p-cresol sulfate	3233-58-7
D-Maltose	69-79-4	Phenaceturic acid	500-98-1
D-Mannose	31103-86-3	Phenyl sulfate	937-34-8
D-pantothenic acid	79-83-4	Phenylacetate	103-82-2
D-Xylose	58-86-6	Phenylpyruvic acid	156-06-9
Glycochenodeoxycholic acid	640-79-9	p-hydroxy-hippuric acid	2482-25-9
Glycodeoxycholic acid	360-65-6	Pyridoxal hydrochloride	65-22-5
Glycoursodeoxycholic acid	64480-66-6	Pyruvic acid	127-17-3
Glyceric acid	473-81-4	Quinic acid	77-95-2
Guanosine	118-00-3	Riboflavin	83-88-5
Hippuric acid	495-69-2	Salicylic acid	69-72-7
Homocitrate	13052-73-8	Serotonin	50-67-9
Hydroxyphenyllactate	306-23-0	Succinic acid	110-15-6
Hypoxanthine	68-94-0	Taurine	107-35-7

Indole lactic acid	1821-52-9	Taurocholic acid	83830-80-2
Indoleacrylic acid	1204-06-4	Taurochenodeoxycholic acid	516-35-8
Indolepropionic acid	830-96-6	Thymine	65-71-4
Indoxyl sulfate	487-94-5	trans-4-Hydroxy-L-proline	51-35-4
Inosine	58-63-9	trans-Aconitic acid	4023-65-8
Itaconic acid	97-65-4	Trehalose	99-20-7
Ketovaleric acid	1821-02-9	Tauroursodeoxycholic acid	14605-22-2
Kynurenic acid	492-27-3	Ursodeoxycholic acid	128-13-2
Lactic acid	50-21-5	Uracil	66-22-8
L-Arabinose	5328-37-0	Uric acid	69-93-2
L-Arabitol	7643-75-6	Uridine	58-96-8
L-asparagine	70-47-3	Vanillic acid	121-34-6
L-Aspartic Acid	56-84-8	Xanthine	69-89-6
L-Carnitine	541-15-1	Xanthosine	146-80-5
L-Citrulline	372-75-8	Xanthurenic acid	59-00-7
L-Glutamic acid	56-86-0	Xylitol	87-99-0

Table S8. Cohort vaccine responder status and percent with 95% confident interval (CI).

Variable		Number "NVR"	% with 95% CI	Number "LVR"	% with 95% CI	Number "none recorded"	% with 95% CI
Sex	Female	27	45% [32.33%,58.31%]	4	33.33% [11.27%,64.56%]	15	51.72% [32.9%,70.11%]
	Male	33	55% [41.69%,67.67%]	8	66.67% [35.44%,88.73%]	14	48.28% [29.89%,67.1%]
Race	Black	5	8.33% [3.11%,19.12%]	1	8.33% [0.44%,40.25%]	3	10.34% [2.71%,28.5%]
	Other	11	18.33% [9.93%,30.85%]	2	16.67% [2.94%,49.12%]	7	24.14% [11.02%,43.93%]
	White/Caucasian	44	73.33% [60.11%,83.55%]	9	75% [42.84%,93.31%]	19	65.52% [45.66%,81.4%]
Delivery Type	C-Section	13	21.67% [12.47%,34.53%]	2	16.67% [2.94%,49.12%]	4	13.79% [4.51%,32.57%]
	Vaginal	47	78.33% [65.47%,87.53%]	10	83.33% [50.88%,97.06%]	25	86.21% [67.43%,95.49%]
Smokers at home?	No	43	71.67% [58.36%,82.18%]	11	91.67% [59.75%,99.56%]	24	82.76% [63.51%,93.47%]
	Yes	9	15% [7.5%,27.08%]	0		4	13.79% [4.51%,32.57%]
	Yes, outside	8	13.33% [6.34%,25.14%]	1	8.33% [0.44%,40.25%]	1	3.45% [0.18%,19.63%]
Siblings	No	22	36.67% [24.89%,50.15%]	4	33.33% [11.27%,64.56%]	8	27.59% [13.45%,47.49%]
	Not Documented	1	1.67% [0.09%,10.14%]	0		0	
	Yes	37	61.67% [48.19%,73.65%]	8	66.67% [35.44%,88.73%]	21	72.41% [52.51%,86.55%]
Pets at home	No	17	28.33% [17.82%,41.64%]	2	16.67% [2.94%,49.12%]	11	37.93% [21.3%,57.64%]
	Yes	43	71.67% [58.36%,82.18%]	10	83.33% [50.88%,97.06%]	18	62.07% [42.36%,78.7%]
Breastfed at enrollment	<50%	2	3.33% [0.58%,12.55%]	2	16.67% [2.94%,49.12%]	4	13.79% [4.51%,32.57%]
	No	10	16.67% [8.7%,28.98%]	0		6	20.69% [8.71%,40.26%]
	Not Documented	1	1.67% [0.09%,10.14%]	0		0	
	Yes,>50%	47	78.33% [65.47%,87.53%]	10	83.33% [50.88%,97.06%]	19	65.52% [45.66%,81.4%]
Breastfed at 6m	No	31	51.67% [38.52%,64.6%]	5	41.67% [16.5%,71.4%]	11	37.93% [21.3%,57.64%]
	Not Documented	3	5% [1.3%,14.82%]	0		9	31.03% [15.98%,50.95%]
	Yes,<50%	2	3.33% [0.58%,12.55%]	1	8.33% [0.44%,40.25%]	1	3.45% [0.18%,19.63%]
	Yes,>50%	24	40% [27.83%,53.45%]	6	50% [25.38%,74.62%]	8	27.59% [13.45%,47.49%]
Daycare at 6m	No	45	75% [61.88%,84.89%]	9	75% [42.84%,93.31%]	15	51.72% [32.9%,70.11%]
	Not Documented	3	5% [1.3%,14.82%]	0		9	31.03% [15.98%,50.95%]
	Yes	12	20% [11.19%,32.7%]	3	25% [6.69%,57.16%]	5	17.24% [6.53%,36.49%]
Family history of ear infections	No	26	43.33% [30.82%,56.7%]	4	33.33% [11.27%,64.56%]	16	55.17% [35.98%,73.05%]
	Yes	34	56.67% [43.3%,69.18%]	8	66.67% [35.44%,88.73%]	13	44.83% [26.95%,64.02%]
Family history of other infections	No	45	75% [61.88%,84.89%]	10	83.33% [50.88%,97.06%]	26	89.66% [71.5%,97.29%]
	Yes	15	25% [15.11%,38.12%]	2	16.67% [2.94%,49.12%]	3	10.34% [2.71%,28.5%]
Family history of atopy	No	34	56.67% [43.3%,69.18%]	4	33.33% [11.27%,64.56%]	14	48.28% [29.89%,67.1%]
	Yes	26	43.33% [30.82%,56.7%]	8	66.67% [35.44%,88.73%]	15	51.72% [32.9%,70.11%]
